# miR-200-mediated inactivation of cancer-associated fibroblasts via targeting of NRP2-VEGFR signaling attenuates lung cancer invasion and metastasis

**DOI:** 10.1016/j.omtn.2024.102194

**Published:** 2024-04-23

**Authors:** Inyoung Cheon, Sieun Lee, Seonyeong Oh, Young-Ho Ahn

**Affiliations:** 1 Department of Molecular Medicine and Inflammation-Cancer Microenvironment Research Center, College of Medicine, Ewha Womans University, Seoul 07804, Korea

**Keywords:** MT: Non-coding RNAs, cancer-associated fibroblasts, invasiveness, lung cancer, miR-200, NRP2

## Abstract

Cancer-associated fibroblasts (CAFs) play a substantial role in promoting cancer cell motility, drug resistance, angiogenesis, and metastasis; therefore, extensive research has been conducted to determine their mode of activation. We aimed to identify whether miRNA-200 (miR-200), a widely recognized suppressor of epithelial-mesenchymal transition, prevents CAFs from promoting cancer progression. Overexpression of miR-200 prevented CAFs from promoting lung cancer cell migration, invasion, tumorigenicity, and metastasis. Additionally, miR-200 suppressed the ability of CAFs to recruit and polarize macrophages toward the M2 phenotype, as well as the migration and tube formation of vascular endothelial cells. NRP2, a co-receptor of vascular endothelial growth factor receptor (VEGFR), was confirmed to be a target of miR-200, which mediates the functional activity of miR-200 in CAFs. NRP2-VEGFR signaling facilitates the secretion of VEGF-D and pleiotrophin from CAFs, leading to the activation of cancer cell migration and invasion. These findings suggest that miR-200 remodels CAFs to impede cancer progression and metastasis and that miR-200 and NRP2 are potential therapeutic targets in the treatment of lung cancer.

## Graphical abstract

**Figure:**
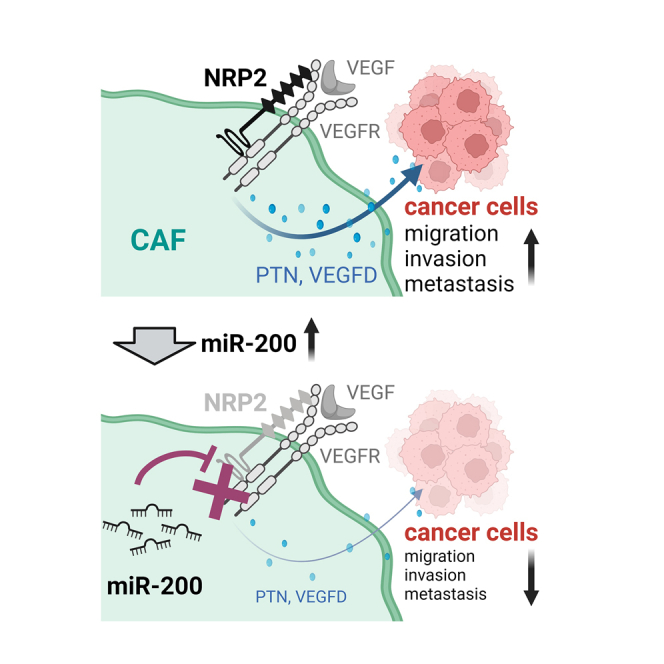

## Teaser

Ahn and colleagues demonstrated that the miR-200 family is downregulated in lung CAFs. miR-200 hinders CAF activation via NRP2/VEGFR signaling, limiting cancer cell mobility. miR-200 also reduces CAF interactions with macrophages and endothelial cells, proposing miR-200 and NRP2 as targets for lung cancer treatment and prognosis.

## Introduction

Cancer-associated fibroblasts (CAFs) are stromal cells found in the connective tissue of solid tumors, including those in lung cancer.^1^ CAFs play a crucial role in the tumor microenvironment (TME) and contribute to the development and progression of lung cancer in several ways.^2^^,^^3^^,^^4^ CAFs secrete growth factors that stimulate angiogenesis, contribute to inflammation, remodel the extracellular matrix (ECM), and modulate immune responses. Further, CAFs directly affect cancer cells by promoting their proliferation, invasion, survival, metastasis, and resistance to chemotherapy.^2^^,^^5^ Therefore, targeting CAFs has gained attention for the improvement of lung cancer therapy. However, a deeper understanding of the mechanisms by which CAFs contribute to lung cancer progression is needed to develop effective treatments.

CAF regulation is influenced by the complex interplay of signals originating from cancer cells and TME. This interplay involves various growth factors and cytokines, including transforming growth factor (TGF)-β, platelet-derived growth factor (PDGF), and IL-1, which modulate CAF behavior in relation to the proliferation, migration, and secretion of matrix-remodeling enzymes and pro-inflammatory cytokines.^6^^,^^7^^,^^8^ The ECM also provides physical and mechanical cues that regulate CAF behavior. Cell-cell interactions with other cells in the TME, including cancer cells, immune cells, and vasculature, further regulate CAF behavior through signaling pathways and paracrine effects.^9^^,^^10^^,^^11^ Additionally, epigenetic modifications, such as DNA methylation and microRNA (miR) expression, play a role in regulating CAF behavior.^2^^,^^12^

miRs are small non-coding RNAs with critical roles in regulating gene expression.^13^ Several studies have identified specific miRs that regulate CAF behavior.^14^ Overexpression of miR-21 in CAFs has been associated with increased proliferation, migration, and secretion of pro-inflammatory cytokines.^15^ miR-29b has been shown to suppress cytokine (CCL11 and CXCL14) production in CAFs and promote breast cancer cell growth and metastasis.^16^ miR-335 reportedly inhibits the migration and activation of hepatic stellate cells and reduces α-smooth muscle actin (α-SMA) and type I collagen production by targeting Tenascin-C.^17^ Previously, we found that CAF activation is facilitated by miR-196a via the regulation of ANXA1 and CCL2, resulting in increased motility and invasiveness of lung cancer cells.^2^ These examples demonstrate the importance of miRs in regulating CAF behavior in the TME and highlight the potential of miRs as a therapeutic target in cancer treatment. This study showed the downregulation of miR-200 family members in CAFs, as reported previously,^2^ and highlighted the significant impact of miR-200 on the interaction between CAFs and cancer cells, leading to the inhibition of lung cancer progression and metastasis.

## Results

### miR-200 inhibits the activation of CAFs

**Figure 1:**
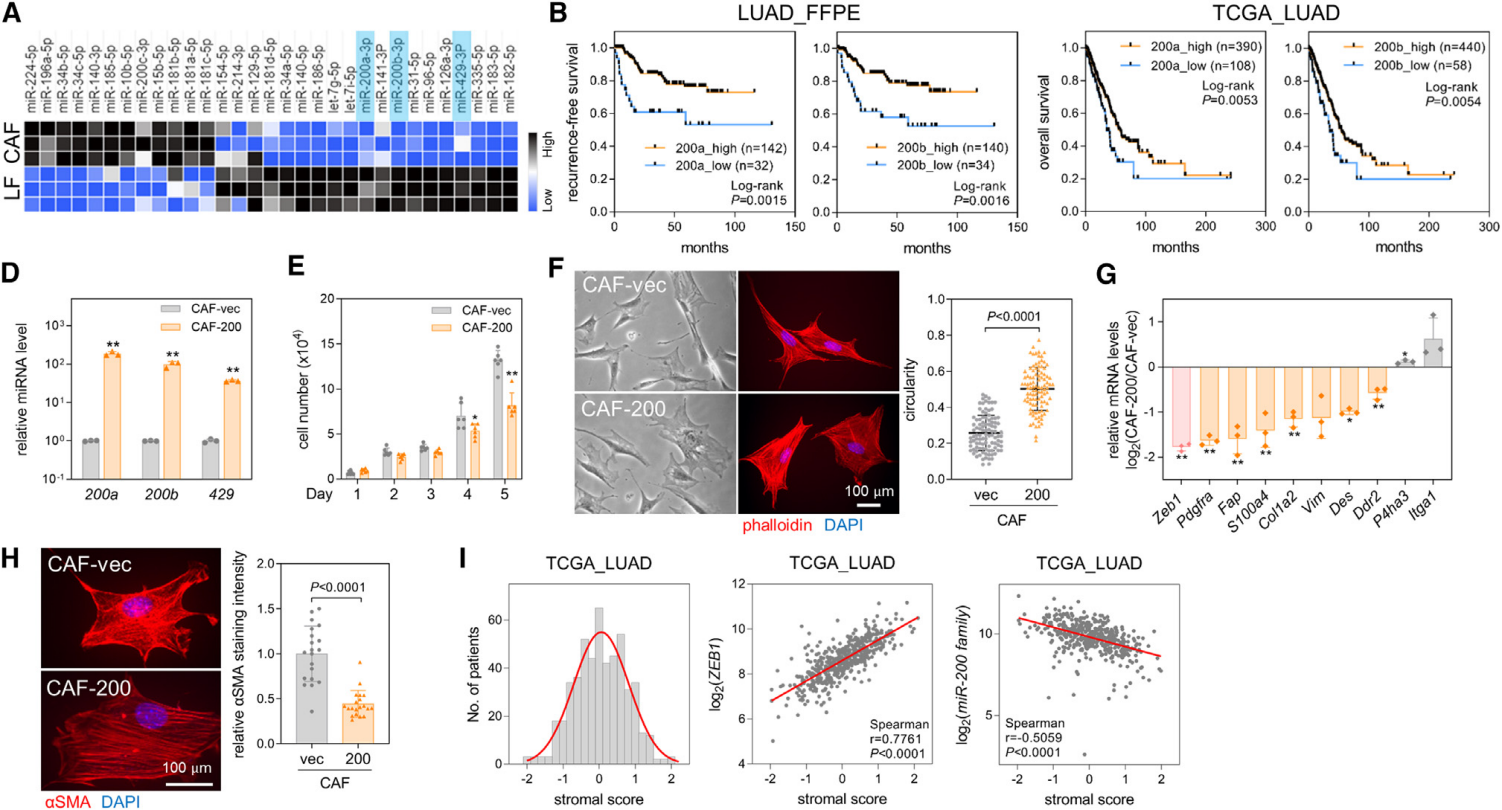
miR-200 family members are downregulated in CAFs (A) A heatmap showing differentially expressed miRs between LFs and CAFs measured using RT-qPCR. Black: increased expression; blue: decreased expression. (B) Kaplan-Meier plots of recurrence-free survival in patients with LUAD. The miR-200a and miR-200b levels were measured by RT-qPCR in FFPE tumor samples from patients with LUAD (*n* = 174). Patients were then classified into two groups (high and low) according to the expression levels of miR-200a and miR-200b. The *p* values were determined by the log rank test. (C) Kaplan-Meier plots of overall survival in patients with LUAD from TCGA data (*n* = 498). Patients were classified into two groups (high and low) based on their miR-200a and miR-200b levels. The *p* values were determined by the log rank test. (D) RT-qPCR of miR-200a, -200b, and -429 in CAFs transduced with empty (CAF-vec) or miR-200 lentiviral vector (CAF-200). miR-200 levels were normalized to the *RNU6B* snoRNA level, and values relative to those of CAF-vec (set at 1.0) are presented. Mean ± SD (*n* = 3). ∗∗*p* < 0.01; two-tailed Student’s *t* test. (E) Cell growth analysis in CAF-vec and CAF-200. Cells were seeded on 12-well plates, and cell numbers were counted on the indicated days after seeding. Data are expressed as mean ± SD (*n* = 6). ∗*p* < 0.05, ∗∗*p* < 0.01; two-tailed Student’s *t* test. (F) Cell morphology of CAF-vec and CAF-200. Representative phase contrast (left) and fluorescence microscopic images (right) are shown. CAF-vec and CAF-200 were stained with phalloidin conjugated to Alexa 594 (red) and DAPI (blue). Circularity was measured using ImageJ. Mean ± SD (CAF-vec, *n* = 100; CAF-200, *n* = 100). *P*, two-tailed Student’s *t* test. (G) RT-qPCR analysis of CAF-specific markers in CAF-vec and CAF-200. The expression was normalized to the *Rpl32* mRNA level, and log_2_ fold-change values (CAF-200 vs. CAF-vec) are presented in the graph. Mean ± SD (*n* = 3). ∗*p* < 0.05, ∗∗*p* < 0.01; two-tailed Student’s *t* test. (H) Immunocytochemistry of α-SMA in CAF-vec and CAF-200. CAF-vec and CAF-200 were subjected to staining using an α-SMA antibody (red) and DAPI (blue). The staining intensity of α-SMA was quantified using ImageJ. Mean ± SD (*n* = 20). *P*, two-tailed Student’s *t* test. (I) A histogram of stromal scores (left) and scatterplots of ZEB1, miR-200 family, and stromal scores (middle and right) obtained from the TCGA-LUAD data. The Spearman correlation coefficient (r) and *p* values are denoted.

Using the NanoString nCounter system, we previously demonstrated that numerous miRs are differentially expressed between normal lung fibroblasts (LFs) and CAFs.^2^ Once confirmed by RT-qPCR, it was observed that among the downregulated miRs in CAFs were miR-200 family members (mir-200a, miR-200b, and miR-429) (Figures 1A and S1A), which are known to suppress epithelial-mesenchymal transition (EMT) in epithelial cancer cells.^18^^,^^19^^,^^20^ Consistent with the fact that EMT is an important process in cancer progression and metastasis,^21^ our analysis of formalin-fixed paraffin-embedded (FFPE) tumor samples from patients with lung adenocarcinoma (LUAD) and The Cancer Genome Atlas (TCGA)-LUAD data revealed that patients with lower miR-200 expression had a relatively poor prognosis compared with those with higher miR-200 expression (Figures 1B and 1C). The majority of research on miR-200 family members in lung cancer has primarily centered on their impacts within cancer cells,^18^^,^^19^^,^^22^ rather than stromal cells like CAFs, prompting our decision to focus on miR-200 in this study. To assess the CAF-specific functions of miR-200, we ectopically overexpressed the miR-200b/a/429 cluster^18^^,^^20^ in murine lung CAFs (Figure 1D). This caused a marginal decrease in cell growth (Figure 1E) and a morphological change characterized by a wider and larger shape resembling that of LFs^2^ (Figure 1F). In addition to decreasing *Zeb1* expression, which is a well-known target of miR-200,^18^ miR-200 overexpression decreased the mRNA levels of most CAF markers^1^ (Figure 1G). miR-200 also inhibited the expression of α-SMA, a marker associated with myofibroblasts (Figure 1H). Analysis of TCGA-LUAD data revealed a negative association between miR-200 expression and stromal scores^23^ (Figure 1I), suggesting that miR-200 hinders desmoplastic lung cancer progression by impeding CAF activation. Further analysis of RNA sequencing data shows that overexpression of miR-200 leads to a decrease in inflammatory CAF markers such as *Cxcl12*, *Clec3b*, *Col14a1*, and *Vegfd*^24^ (Figure S1B). Transfection of a miR-200b inhibitor into LFs enhanced both myofibroblastic and inflammatory CAF markers (Figure S1C). These results suggest that miR-200 suppresses both myofibroblastic and inflammatory features of CAFs. Rank-rank hypergeometric overlap analysis with RNA sequencing data from normal LFs versus CAFs^2^ and CAFs overexpressing miR-200 (CAF-200) versus control CAFs reveals a significant overlap of downregulated genes between CAF-200 and LFs (Figure S1D). This implies that miR-200 suppresses common CAF features and promotes the reprogramming of CAFs into LFs.

### CAFs reprogrammed by miR-200 have a poor ability to activate lung cancer cells

**Figure 2:**
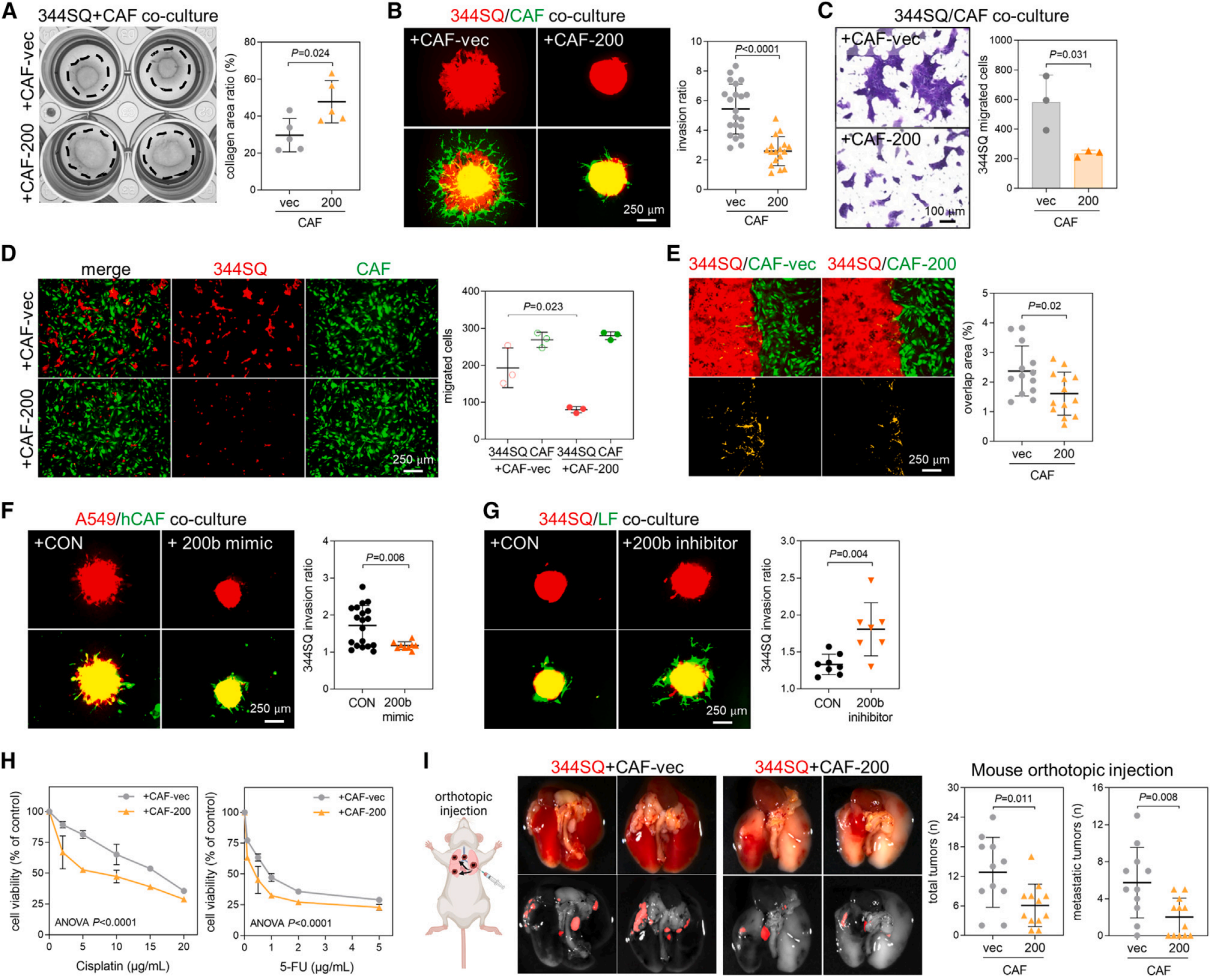
miR-200 prevents CAFs from facilitating cancer cell migration and invasion (A) Collagen gel contraction assay of 344SQ cells co-cultured with CAF-vec and CAF-200. Collagen area was measured using ImageJ. Mean ± SD (*n* = 5). *P*, two-tailed Student’s *t* test. (B) Spheroid invasion assay of 344SQ cells co-cultured with CAF-vec or CAF-200. The 344SQ cells were labeled with mCherry (red fluorescence), and fibroblasts were labeled with GFP. Spheroids made from hanging-drop cultures were seeded on collagen gels and cultured for 2 days. Spheroid invasion ratios (ratio of whole cell area to central spheroid area) were measured using ImageJ. Mean ± SD (+CAF-vec, *n* = 21; +CAF-200, *n* = 17). *P*, two-tailed Student’s *t* test. (C) Transwell migration assay of 344SQ cells co-cultured with CAF-vec or CAF-200. Fibroblasts were seeded in the bottom wells, and 344SQ cells were seeded in the upper wells. After 24 h, the migrated 344SQ cells were stained with crystal violet, photographed, and counted. Mean ± SD (*n* = 3). *P*, two-tailed Student’s *t* test. (D) Transwell migration assay of 344SQ cells co-cultured with CAF-vec and CAF-200. Both 344SQ cells and CAF-vec or CAF-200 were seeded together in the upper wells. After 24 h, the migrated 344SQ cells (red) and fibroblasts (green) were photographed and counted. Mean ± SD (*n* = 3). *P*, two-tailed Student’s *t* test. (E) Two-well migration assay of 344SQ with CAF-vec and CAF-200. The 344SQ and CAF-vec or CAF-200 were seeded separately on each side of the Culture-Insert 2 Well. When the cells grew confluently, the wells were removed to allow them to migrate toward each other. After 24 h, overlap areas were photographed and measured by ImageJ. Mean ± SD (*n* = 13). *P*, two-tailed Student’s *t* test. (F) Spheroid invasion assay of A549 cells (labeled with mCherry) co-cultured with human CAFs (labeled with CellTracker Green) transfected with the control (+CON) or miR-200b mimic (+200b mimic). Mean ± SD (+CON, *n* = 19; +200b mimic, *n* = 9). *P*, two-tailed Student’s *t* test. (G) Spheroid invasion assay of 344SQ cells co-cultured with murine normal LFs transfected with control (+CON) or miR-200b inhibitor (+200b inhibitor). Mean ± SD (+CON, *n* = 8; +200b inhibitor, *n* = 7). *P*, two-tailed Student’s *t* test. (H) Cell viability of 344SQ cancer cells treated with cisplatin or 5-FU in the presence of CAF-vec or CAF-200. The 344SQ cells were co-cultured with CAF-vec or CAF-200 and treated with cisplatin (0–20 μM) or 5-FU (0–5 μg/mL). After 48 h, the 344SQ cells and CAFs were photographed under a fluorescence microscope, and the density of 344SQ cells was measured using ImageJ. Mean ± SD (*n* = 6). *P*, two-way ANOVA. (I) Orthotopic injection of 344SQ cells (5 × 10^5^ cells/mouse, labeled with mCherry) with CAF-vec or CAF-200 (5 × 10^5^ cells/mouse) into syngeneic mice (129/Sv). After 10 days, the mice were necropsied, and the lungs were photographed. The graphs show the total and metastatic tumors. Mean ± SD (+CAF-vec, *n* = 11; +CAF-200, *n* = 12). *P*, two-tailed Student’s *t* test.

To explore the effect of miR-200 on the ability of CAFs to activate cancer cells, we used various co-culture models of CAFs and lung cancer cells. In the collagen gel contraction assay, miR-200 suppressed the contractility of CAFs co-cultured with murine lung cancer cells (344SQ, 344LN, and 531LN2) derived from *Kras/Trp53*-mutant mice^25^ (Figures 2A, S2A, and S2D). Spheroids with CAF-200 and cancer cells showed reduced invasiveness in collagen gels compared with those with CAF-vec and cancer cells (Figures 2B, S2B, and S2E). In the transwell migration assay, neither CAF-200 cultured separately on the bottom nor together on the upper inserts promoted cancer cell migration compared with CAF-vec (Figures 2C, 2D, S2C, and S2F). The ability of CAFs to interact with cancer cells was also reduced after miR-200 overexpression in the two-well migration assay (Figure 2E). Human lung CAFs transfected with the miR-200b mimic attenuated cancer spheroid invasion (Figures 2F and S2G), whereas LFs transfected with the miR-200b inhibitor restored cancer spheroid invasion (Figures 2G and S2H). Among other members of the miR-200 family, miR-429 displayed suppressive effects (Figure S2I), whereas its inhibitor augmented CAF activity (Figure S2J). However, the impact of the miR-200a mimic and inhibitor was relatively weaker compared with miR-200b and miR-429 (Figures S2K and S2L). This suggests that miR-200b and miR-429, which share identical seed sequences, are the main miRs regulating CAF activity within the miR-200b/a/429 cluster. Moreover, cancer cells co-cultured with CAF-200 exhibited greater sensitivity to anticancer drugs, cisplatin and 5-fluorouracil (5-FU), than those co-cultured with CAF-vec (Figures 2H and S3A), indicating that miR-200 decreases the ability of CAFs to enhance drug resistance in cancer cells. When 344SQ lung cancer cells with CAF-200 or CAF-vec were injected into 129/Sv syngeneic mice^26^ orthotopically in the lung, subcutaneously in the flank, or intravenously via the lateral tail veins, CAF-200 formed fewer metastatic nodules than did CAF-vec (Figures 2I, S3B, and S3C). In the subcutaneous injection model, the impact of miR-200 on drug resistance was examined. Consistent with the *in vitro* findings from co-cultures of 344SQ and CAFs, tumors harboring CAF-200 exhibited greater sensitivity to 5-FU compared with those with CAF-vec; notably, 5-FU treatment significantly inhibited tumor growth in the presence of CAF-200 compared with control conditions (Figure S3D). Therefore, miR-200 triggers the reprogramming of CAFs, which decreases their ability to facilitate the migration, invasion, and drug resistance of lung cancer cells.

### NRP2 is a target of miR-200

**Figure 3:**
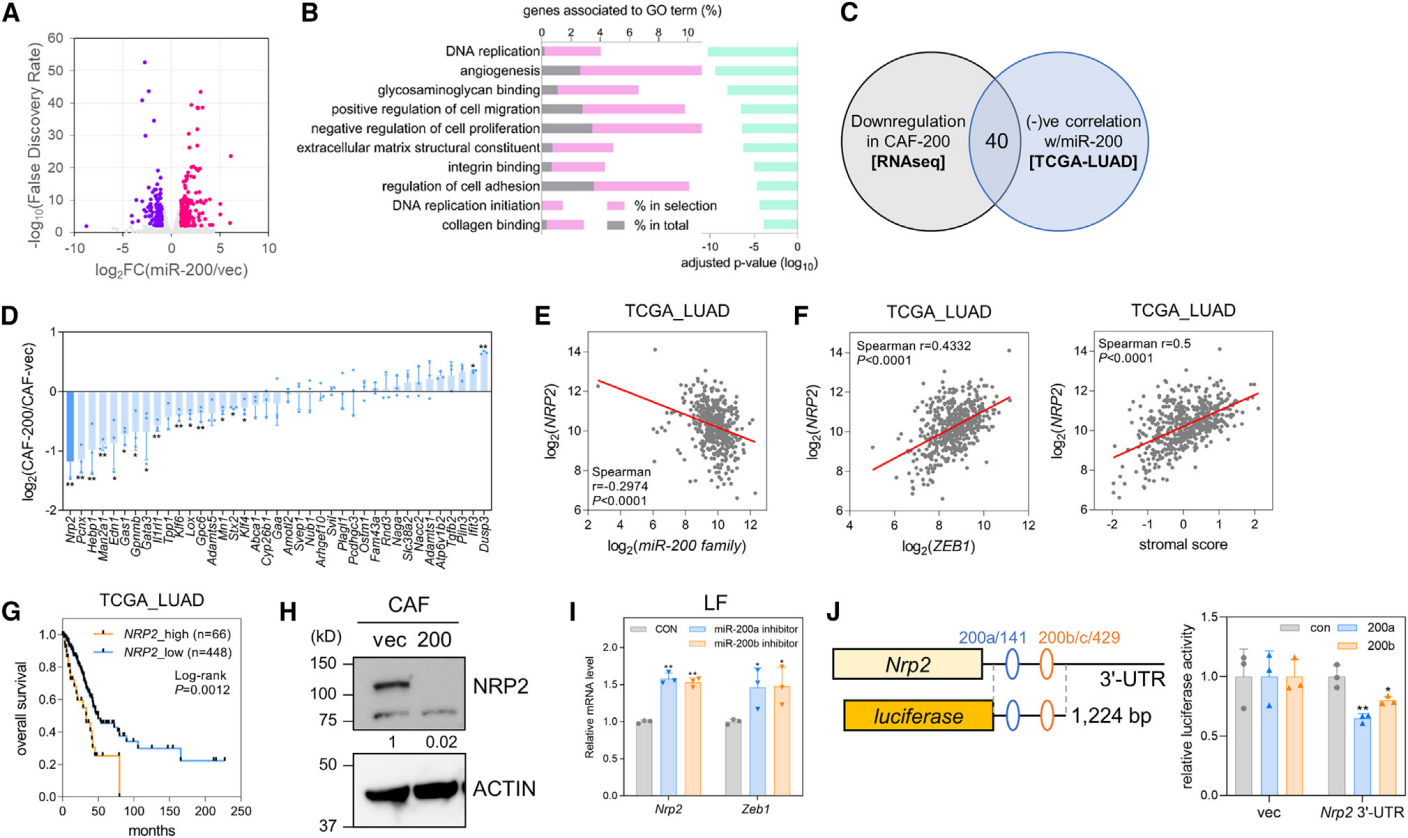
*Nrp2* is a target of miR-200 (A) A volcano plot of RNA sequencing data in CAF-vec and CAF-200. Purple, downregulated; pink, upregulated genes in CAF-200 vs. CAF-vec (log_2_ fold-change >1, false discovery rate < 0.01). (B) Gene ontology (GO) enrichment analysis of 347 genes downregulated in CAF-200 compared with CAF-vec using Metascape (https://metascape.org). Genes associated with the GO terms are shown in the left bar graph for the upregulated genes (% in selection, pink) and for the total set of 20,611 genes (% in total, gray). The adjusted *p* values are represented in the right bar graph (green). (C) A Venn diagram showing the prediction of miR-200 target genes through RNA sequencing and TCGA-LUAD data. (D) RT-qPCR analysis of predicted target genes of miR-200 in CAF-vec and CAF-200. The expression was normalized to the *Rpl32* mRNA level, and log_2_ fold-changes (CAF-200 vs. CAF-vec) are presented in the graph. Mean ± SD (*n* = 3). ∗*p* < 0.05, ∗∗*p* < 0.01; two-tailed Student’s *t* test. (E) Correlation between miR-200 family and *NRP2* expression levels in TCGA-LUAD data. Spearman correlation coefficient (r) and *p* value are shown in the graph. (F) Correlation between *NRP2* and *ZEB1* expression levels (left) or stromal scores (right) in TCGA-LUAD data. Spearman correlation coefficient (r) and *p* value are denoted. (G) Kaplan-Meier plots of overall survival in patients with LUAD from TCGA data (*n* = 514). Patients were classified into two groups (high and low) based on their *NRP2* mRNA levels. *p* values were determined by the log rank test. (H) Western blot of NRP2 protein in CAF-vec and CAF-200. Actin was used as a control. Relative quantitation results are shown below. (I) RT-qPCR analysis of *Nrp2* and *Zeb1* in LFs transfected with miR-200a and 200b inhibitors. The expression was normalized to the *Rpl32* mRNA level. Mean ± SD (*n* = 3). ∗*p* < 0.05, ∗∗*p* < 0.01; two-tailed Student’s *t* test. (J) Luciferase reporter assay with *Nrp2* 3′-UTR. Part of the *Nrp2* 3′-UTR (1,224 bp) containing putative miR-200-binding sites (predicted using TargetScan) was cloned into pGL3-Basic vector and then co-transfected with miR-200 mimics (200a and 200b) or control into 293T cells. Mean ± SD (*n* = 3). ∗*p* < 0.05, ∗∗*p* < 0.01; two-tailed Student’s *t* test.

To identify the downstream targets of miR-200 that mediate its role in CAFs, we performed transcriptomic profiling using bulk RNA sequencing of CAF-vec and CAF-200 cells (Figure 3A). Gene ontology analysis of the downregulated genes in CAF-200 revealed that miR-200 inhibited the expression of genes associated with angiogenesis, cell migration, cell adhesion, ECM, and integrin/collagen (Figure 3B). Among these downregulated genes, we selected 40 genes, the expression of which was negatively correlated with that of miR-200, from the TCGA-LUAD data (Figure 3C), and measured the mRNA levels in CAF-vec and CAF-200 using RT-qPCR (Figure 3D). NRP2, which is a co-receptor for vascular endothelial growth factor receptor (VEGFR), c-Met, PDGF receptor (PDGFR), TGF-βR, and integrins and regulates angiogenesis, invasion, and metastasis,^27^ was the most decreased by miR-200 (Figure 3D). *NRP2* expression was negatively correlated with miR-200 family members (Figures 3E and S4A) and positively correlated with *ZEB1* or stromal scores in TCGA-LUAD data (Figure 3F). In addition, high *NRP2* expression was associated with a poor prognosis in patients with LUAD, according to the TCGA data (Figure 3G). NRP2 protein expression decreased after miR-200 overexpression (Figures 3H and S4B), *Nrp2* mRNA expression increased after the treatment of miR-200 inhibitors (Figure 3I), and *Nrp2* 3′-UTR reporter activity was suppressed by miR-200 (Figure 3J), providing further evidence that NRP2 is a direct target of miR-200 in CAFs.

### NRP2 serves as a critical mediator of CAF activity inhibition by miR-200

**Figure 4:**
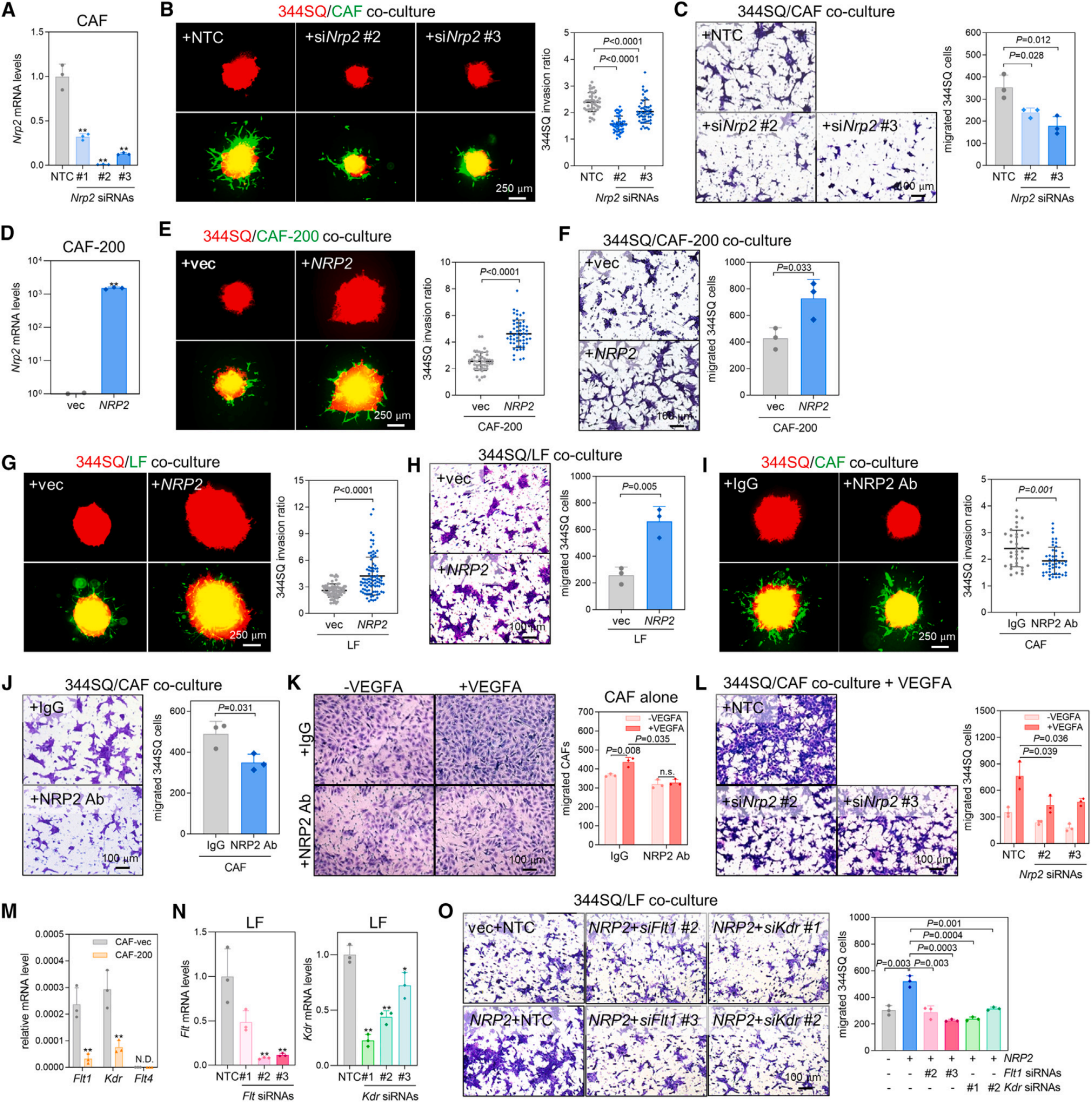
NRP2 mediates the inhibition of CAF activity by miR-200 (A) RT-qPCR analysis of *Nrp2* expression in murine CAF transfected with non-targeting control (NTC) or *Nrp2* siRNAs (#1, #2, and #3). The expression levels were normalized to the *Rpl32* mRNA level, and values relative to that of NTC (set at 1.0) are presented. Mean ± SD (*n* = 3). *P*, two-tailed Student’s *t* test. (B) Spheroid invasion assay of 344SQ cells co-cultured with CAF transfected with NTC or *Nrp2* siRNAs (si*Nrp2*). Mean ± SD (+NTC, *n* = 41; +si*Nrp2* #2, *n* = 48; +si*Nrp2* #3, *n* = 52). *P*, two-tailed Student’s *t* test. (C) Transwell migration assay of 344SQ cells co-cultured with CAF transfected with NTC or si*Nrp2*. CAFs transfected with NTC or si*Nrp2* were seeded in the bottom wells, and 344SQ cells were seeded in the upper inserts. After 24 h, the migrated 344SQ cells were photographed and counted. Mean ± SD (*n* = 3). *P*, two-tailed Student’s *t* test. (D) RT-qPCR analysis of *Nrp2* in CAF-200 transfected with an empty vector (+vec) or a *NRP2*-overexpressing vector (+*NRP2*). Mean ± SD (*n* = 3). *P*, two-tailed Student’s *t* test. (E) Spheroid invasion assay of 344SQ cells co-cultured with CAF-200 transfected with control or *NRP2* overexpressing vector. Mean ± SD (+vec, *n* = 45; +*NRP2*, *n* = 54). *P*, two-tailed Student’s *t* test. (F) Transwell migration assay of 344SQ cells co-cultured with CAF-200 transfected with control or *NRP2* overexpressing vector. CAF-200 transfected with control or *NRP2* overexpressing vector were seeded in the bottom wells, and 344SQ cells were seeded in the upper inserts. After 24 h, the migrated 344SQ cells were photographed and counted. Mean ± SD (*n* = 3). *P*, two-tailed Student’s *t* test. (G) Spheroid invasion assay of 344SQ cells co-cultured with LFs transfected with *NRP2* (LF + *NRP2*) or empty vector (LF + vec). Mean ± SD (+vec, *n* = 70; +*NRP2*, *n* = 91). *P*, two-tailed Student’s *t* test. (H) Transwell migration assay of 344SQ cells co-cultured with LF + vec and LF + *NRP2*. Mean ± SD (*n* = 3). *P*, two-tailed Student’s *t* test. (I) Spheroid invasion assay in 344SQ cells co-cultured with CAF in the presence (+NRP2 Ab) or absence (+IgG) of NRP2 neutralizing Ab (1 μg/mL). Mean ± SD (+IgG, *n* = 34; +NRP2 Ab, *n* = 49). *P*, two-tailed Student’s *t* test. (J) Transwell migration assay of 344SQ cells co-cultured with CAF in the presence or absence of NRP2 Ab. 344SQ cells were seeded on the upper wells, and fibroblasts were seeded in the bottom wells. After 24 h, the migrated 344SQ cells were photographed and counted. Mean ± SD (*n* = 3). *P*, two-tailed Student’s *t* test. (K) Transwell migration assay of CAFs treated with NRP2 Ab (1 μg/mL) and VEGF-A recombinant protein (20 ng/mL). After 24 h, migrated CAFs were photographed and counted. Mean ± SD (*n* = 3). *P*, two-tailed Student’s *t* test. (L) Transwell migration assay of 344SQ cells co-cultured with CAFs transfected with NTC or *Nrp2* siRNAs. 344SQ cells were seeded on the upper wells, and CAFs were seeded in the bottom wells in the presence or absence of VEGF-A. After 24 h, the migrated 344SQ cells were photographed and counted. Mean ± SD (*n* = 3). *P*, two-tailed Student’s *t* test. (M) RT-qPCR analysis of *Flt1*, *Kdr*, and *Flt4* in CAF-vec and CAF-200. Expression levels were normalized to the *Rpl32* mRNA level. Mean ± SD (*n* = 3). *P*, two-tailed Student’s *t* test. N.D., not detected. (N) RT-qPCR analysis of *Flt1* (left) and *Kdr* (right) of LFs transfected with NTC, *Flt1* siRNAs or *Kdr* siRNAs (#1, #2, and #3). Mean ± SD (*n* = 3). *P*, two-tailed Student’s *t* test. (O) Transwell migration assay in 344SQ cells co-cultured with LFs transfected with *NRP2* overexpressing vector and siRNAs against *Flt1* or *Kdr*. LFs transfected with *NRP2* and siRNAs were seeded in the bottom wells, and 344SQ cells were seeded in the upper inserts. After 24 h, the migrated 344SQ cells were photographed and counted. Mean ± SD (*n* = 3). *P*, two-tailed Student’s *t* test.

Next, we attempted to elucidate the function of NRP2 in CAFs. *Nrp2* knockdown by small interfering RNAs (siRNAs) (Figure 4A) decreased the capacity of CAFs to promote cancer cell invasion and migration (Figures 4B and 4C). Conversely, the add-back of NRP2 to CAF-200 cells (Figures 4D and S5A) restored their capacity to promote cancer cell invasion and migration (Figures 4E and 4F), and NRP2 overexpression enhanced the ability of LFs to promote cancer cell invasion and migration (Figures 4G and 4H). In addition, the NRP2-blocking antibody prevented CAFs from promoting cancer cell invasion and migration (Figures 4I and 4J). Comparable with CAF-200, NRP2-depleted CAFs tended to lack the ability to facilitate the metastatic colonization of 344SQ cells in a mouse tail vein injection model (Figures S5B and S5C), indicating that NRP2 is essential for the interaction of CAFs with cancer cells. As mentioned above, NRP2 is a co-receptor of VEGFR that mediates VEGF signaling.^27^ VEGF-A stimulated the migration of CAFs and cancer cells, which was blocked by an NRP2 blocking antibody (Figure 4K) and *Nrp2* siRNAs (Figure 4L). Among the three VEGFRs (VEGFR1–3), VEGFR1 (encoded by *Flt1*) and VEGFR2 (encoded by *Kdr*) were detectable in CAFs and were downregulated by miR-200 (Figure 4M), consistent with previous findings that identified them as miR-200 targets.^19^^,^^28^ The knockdown of *Flt1* and *Kdr* in LFs (Figure 4N) resulted in a decrease in cancer cell migration and invasion enhanced by NRP2 (Figures 4O and S5D), indicating the essential role of VEGFR1 and VEGFR2 in the NRP2-mediated regulation of CAF activation. These results suggest that miR-200 inhibits CAF activity by targeting NRP2/VEGFR signaling.

### CAF-derived VEGF-D and pleiotrophin activate cancer cell migration and invasion

**Figure 5:**
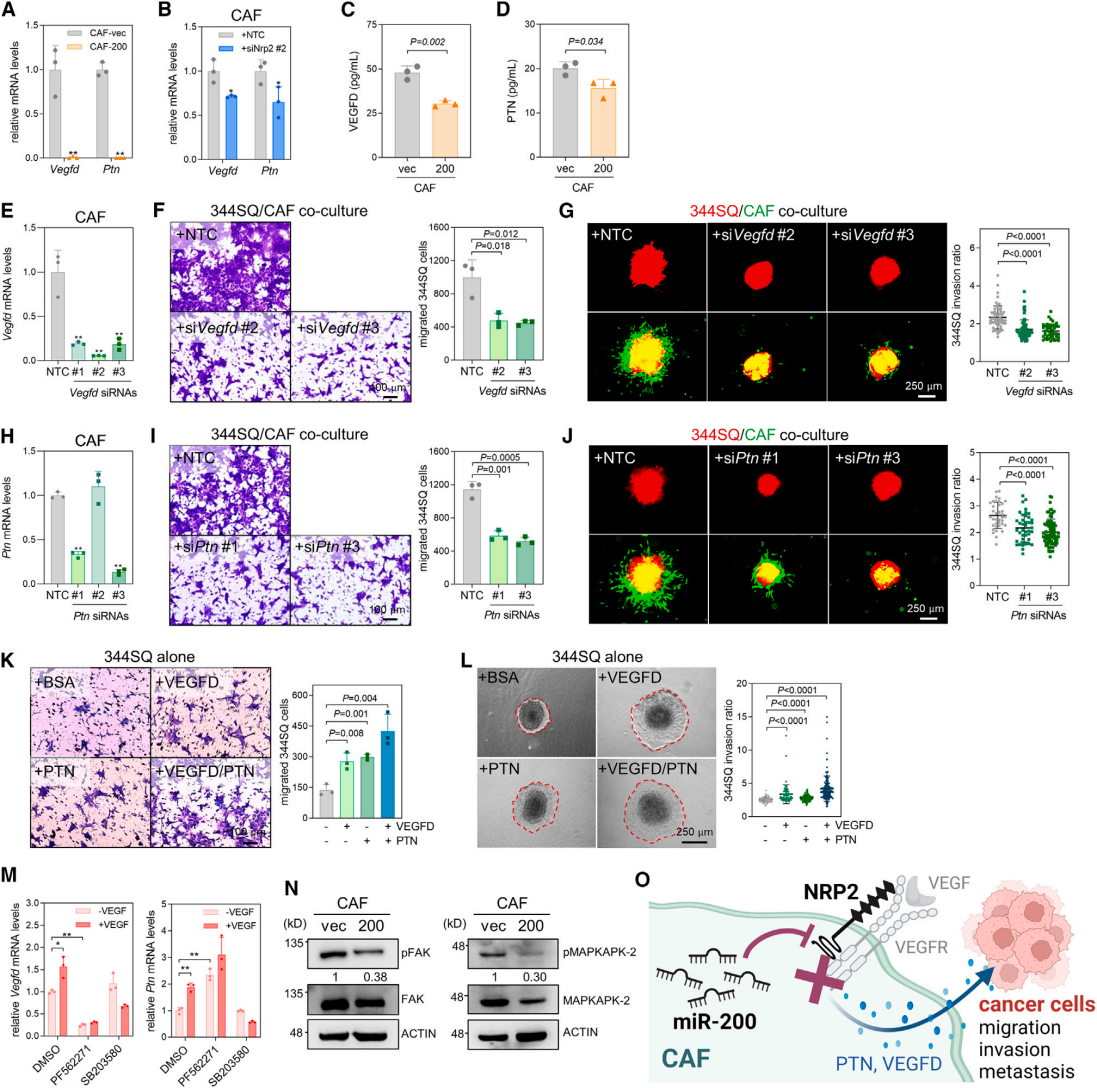
VEGF-D and PTN derived from CAFs activate cancer cell migration and invasion (A and B) RT-qPCR analysis of *Vegfd* and *Ptn* expression in CAF-vec and CAF-200 (A), and CAFs transfected with NTC or *Nrp2* siRNA #2 (*siNrp2* #2) (B). The expression levels were normalized to the *Rpl32* mRNA level, and the values relative to those of the control (set at 1.0) are presented. Mean ± SD (*n* = 3). *P*, two-tailed Student’s *t* test. (C, D) ELISA of secreted VEGF-D (C) and PTN (D) in the CAF-vec and CAF-200 culture media. Mean ± SD (*n* = 3). *P*, two-tailed Student’s *t* test. (E) RT-qPCR analysis of *Vegfd* expression in murine CAFs transfected with NTC or *Vegfd* siRNAs (#1, #2, and #3). The expression levels were normalized to the *Rpl32* mRNA level, and the values relative to those of NTC (set at 1.0) are presented. Mean ± SD (*n* = 3). *P,* two-tailed Student’s *t* test. (F) Transwell migration assay of 344SQ cells co-cultured with CAFs transfected with NTC or *Vegfd* siRNAs (si*Vegfd* #2 and #3). CAFs were seeded in the bottom wells, and 344SQ cells were seeded in the upper wells. After 24 h, the migrated 344SQ cells were photographed and counted. Mean ± SD (*n* = 3). *P*, two-tailed Student’s *t* test. (G) Spheroid invasion assay of 344SQ cells co-cultured with CAFs transfected with NTC or si*Vegfd*. Mean ± SD (+NTC, *n* = 59; +si*Vegfd* #2, *n* = 73; +si*Vegfd* #3, *n* = 45). *P*, two-tailed Student’s *t* test. (H) RT-qPCR analysis of *Ptn* expression in murine CAFs transfected with NTC or *Ptn* siRNAs (#1, #2, and #3). Mean ± SD (*n* = 3). *P*, two-tailed Student’s *t* test. (I) Transwell migration assay of 344SQ cells co-cultured with CAFs transfected with NTC or *Ptn* siRNAs (si*Ptn* #1 and #3). Mean ± SD (*n* = 3). *P*, two-tailed Student’s *t* test. (J) Spheroid invasion assay in 344SQ cells co-cultured with CAFs transfected with NTC or si*Ptn*. Mean ± SD (+NTC, *n* = 37; +si*Ptn* #1, *n* = 41; +si*Ptn* #3, *n* = 78). *P*, two-tailed Student’s *t* test. (K) Transwell migration assay of 344SQ cells treated with VEGF-D and PTN recombinant proteins. 344SQ cells were seeded in the upper inserts and VEGF-D and PTN recombinant proteins (20 ng/mL) were added to the bottom wells. After 24 h, migrated 344SQ cells were photographed and counted. Mean ± SD (*n* = 3). *P*, two-tailed Student’s *t* test. (L) Spheroid invasion assay of 344SQ cells treated with recombinant VEGF-D and PTN. Mean ± SD (+BSA, *n* = 67; +VEGF-D, *n* = 73; +PTN, *n* = 70; +VEGF-D/PTN, *n* = 149). *P*, two-tailed Student’s *t* test. (M) RT-qPCR analysis of *Vegfd* and *Ptn* expression in CAFs treated with VEGF-A plus inhibitors of FAK (PF-562271, 10 μM) and p38 MAPK (SB203580, 10 μM). Mean ± SD (*n* = 3). ∗*p* < 0.05, ∗∗*p* < 0.01; two-tailed Student’s *t* test. (N) Western blots of phospho-FAK, FAK, phospho-MAPKAPK2, and MAPKAPK2 (a substrate of p38 MAPK) in CAF-vec and CAF-200. Actin was used as a control. Relative quantitation results (phospho-form/total) are shown below. (O) A diagram suggesting that miR-200 suppresses CAF-derived VEGF-D and PTN secretion by targeting NRP2/VEGFR signaling, leading to the inhibition of cancer cell migration and invasion.

CAFs secrete diverse cytokines and growth factors that modulate cancer cell proliferation, migration, invasion, drug resistance, and metastasis.^29^ Given that miR-200 modulates the cancer cell secretome, including VEGF signaling,^19^^,^^30^ we examined the RNA sequencing data thoroughly and found that many cytokines and growth factors were downregulated in CAF-200 cells compared with CAF-vec cells (Figure S6A). Pleiotrophin (PTN) and VEGF-D, which are closely related to NRP2 and VEGF-A in the STRING database (Figure S6B), were among the most downregulated genes in CAF-200 cells (Figure S6C). Both mRNA and protein levels of VEGF-D and PTN decreased with miR-200 overexpression and *Nrp2* knockdown (Figures 5A–5D), and reportedly promoted the migration and metastasis of lung cancer cells.^31^^,^^32^ Hence, we surmised that these two growth factors are interactive mediators between CAFs and cancer cells controlled by the miR-200 and NRP2 axis. Indeed, *Vegfd* and *Ptn* knockdown in CAFs by siRNAs (Figures 5E and 5H) inhibited cancer cell migration (Figures 5F and 5I) and invasion in co-culture models (Figures 5G and 5J). Conditioned media (CM) from *Vegfd-* and *Ptn-*knockdown CAFs suppressed cancer cell migration and invasion (Figures S6D–S6G). Additionally, treatment with recombinant VEGF-D and PTN proteins promoted the migration and invasion of lung cancer cells (Figures 5K and 5L). *Vegfd* and *Ptn* mRNA levels induced by VEGF-A were attenuated by inhibitors of FAK and p38 MAPK (Figure 5M), known as downstream mediators of NRP2/VEGFR signaling.^27^ Indeed, FAK and p38 MAPK were inactivated in CAF-200 compared with CAF-vec (Figure 5N). These data demonstrate that miR-200 suppresses CAF-derived VEGF-D and PTN secretion by targeting NRP2/VEGFR signaling, leading to the inhibition of cancer cell invasiveness (Figure 5O).

### miR-200 attenuates the activity of CAFs to interact with macrophages and vascular endothelial cells

**Figure 6:**
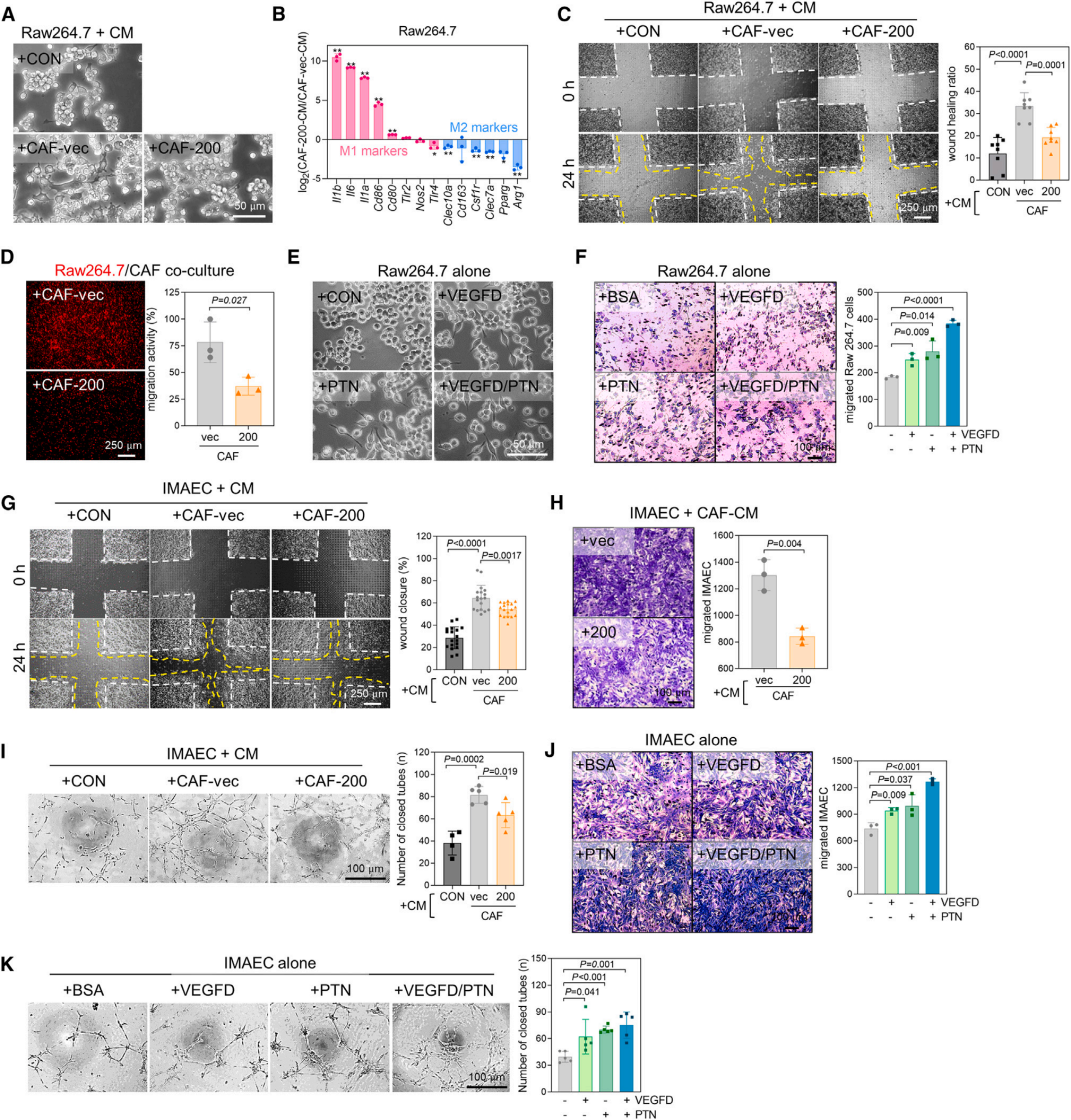
miR-200 attenuates CAF activity to recruit macrophages and vascular endothelial cells (A) Phase-contrast microscopic images of Raw264.7 cells treated with CM of CAF-vec and CAF-200. (B) RT-qPCR analysis of M1 and M2 macrophage markers in Raw264.7 cells treated with CM of CAF-vec and CAF-200. The expression was normalized to the *Rpl32* mRNA level, and log_2_ fold-change values (CAF-200 vs. CAF-vec) are presented in the graph. Mean ± SD (*n* = 3). ∗*p* < 0.05, ∗∗*p* < 0.01; two-tailed Student’s *t* test. (C) Wound healing assay of Raw264.7 cells treated with CM of CAF-vec and CAF-200. Wound closure (1 − [wound area ratio of 24 h to 0 h]) was measured using ImageJ. Mean ± SD (*n* = 8). *P*, two-tailed Student’s *t* test. (D) Transwell migration assay of Raw264.7 cells (labeled with CellTracker Red) treated with CM of CAF-vec and CAF-200. Mean ± SD (*n* = 3). *P*, two-tailed Student’s *t* test. (E) Phase-contrast microscopic images of Raw264.7 cells treated with VEGF-D and PTN recombinant proteins. (F) Transwell migration assay of Raw264.7 cells treated with VEGF-D and PTN. Mean ± SD (*n* = 3). *P*, two-tailed Student’s *t* test. (G) Wound healing assay of IMAEC treated with CM of CAF-vec and CAF-200. Mean ± SD (*n* = 18). *P*, two-tailed Student’s *t* test. (H) Transwell migration assay of IMAEC treated with CM of CAF-vec and CAF-200. Mean ± SD (*n* = 3). *P*, two-tailed Student’s *t* test. (I) Tube formation assay of IMAEC treated with CM of CAF-vec and CAF-200. The graph shows the counts of enclosed tubes in each microscopic field. Mean ± SD (+CON, *n* = 4; CAF+vec, *n* = 5; CAF+200, *n* = 5). *P*, two-tailed Student’s *t* test. (J) Transwell migration assay of IMAEC treated with VEGF-D and PTN. Mean ± SD (*n* = 3). *P*, two-tailed Student’s *t* test. (K) Tube formation assay of IMAEC treated with VEGF-D and PTN. Mean ± SD (*n* = 5). *P*, two-tailed Student’s *t* test.

Through interaction with other tumor-associated stromal cells, such as macrophages and vascular endothelial cells, CAFs play a crucial role in the TME in promoting tumor growth and progression.^33^ CAFs recruit monocytes and induce M2 polarization, which creates an anti-inflammatory and pro-tumorigenic environment.^9^^,^^34^ Raw264.7 murine macrophages treated with CM from CAF-vec were polarized into M2 macrophages, whereas those treated with CAF-200 CM were polarized into M1 macrophages (Figures 6A and 6B; Figures S7A and S7B). Furthermore, CAF-200 showed a reduced ability to promote macrophage migration (Figures 6C and 6D). VEGF-D and PTN recombinant proteins also promoted macrophage migration (Figures 6E and 6F). CAFs activate vascular endothelial cells (immortalized mouse aortic endothelial cells [IMAEC]) and facilitate angiogenesis.^11^ Unlike CAF-vec CM, CAF-200 CM did not promote IMAEC migration (Figures 6G and 6H) or tube formation (Figure 6I). VEGF-D and PTN promoted IMAEC migration and tube formation (Figures 6J and 6K). In mouse lung tumors, CAF-vec facilitated macrophage infiltration, M2 polarization, and angiogenesis (Figures S7C and S7D). These results indicate that miR-200 suppresses CAF activity to crosstalk with macrophages and vascular endothelial cells in the TME.

## Discussion

The miR-200 family of miRs, which is recognized for its ability to suppress EMT,^18^^,^^35^ regulates the interaction between CAFs and cancer cells in the TME. In a LUAD mouse model, miR-200 knockout enhanced the interaction between CAFs and cancer cells by de-repressing Jagged1 and Jagged2 in cancer cells, leading to enhanced invasion and metastasis.^30^ In co-cultures with gastric cancer cells, CAFs induce hypermethylation on the promoter of the miR-200b/200a/429 cluster and suppress miR-200 expression in cancer cells, eventually enhancing migration and invasion.^36^ Although numerous studies have investigated the roles of the miR-200 family members in cancer cells, several others have explored their functions in CAFs. miR-200 was found to inhibit CAF activation and ECM remodeling by targeting *FLI1* and *TCF12*, thereby interfering with the invasion and metastasis of breast cancer cells.^37^ Colon cancer cells co-cultured with miR-200c-overexpressing CAFs showed decreased invasiveness.^38^ However, despite substantial research efforts, the exact functional effects and mechanisms by which miR-200 modulates CAF activity in the lung TME remain unclear. Here, we showed that miR-200 inhibited CAF activation by targeting NRP2/VEGFR signaling, decreasing the aggressiveness of lung cancer cells, preventing M2 polarization of macrophages, and suppressing the angiogenic sprouting of vascular endothelial cells.

CAF activation is induced by extrinsic factors from the TME^9^^,^^10^ and intrinsic factors within CAFs.^2^^,^^39^ miR-200 overexpression suppressed the expression of CAF activation markers (e.g., α-SMA, PDGFα, FAP, FSP1, type I collagen α2, vimentin, and desmin), and miR-200 expression negatively correlated with stromal scores in TCGA-LUAD data, showing that miR-200 inhibits CAF activation. The ability of miR-200 to suppress CAF activation can be attributed to its ability to target ECM structural and cell adhesion proteins.^40^ Our RNA sequencing data also revealed that miR-200 downregulates CAF features associated with ECM remodeling, cell adhesion, and collagen-integrin binding, which requires further validation.

Vascularization and angiogenesis are the signaling pathways targeted by miR-200.^41^ VEGF is directly targeted by miR-200 family members in diabetic retinopathy,^42^ chondrosarcoma,^43^ and lung cancer.^44^ FLT1/VEGFR1 and KDR/VEGFR2 were also confirmed as direct targets of miR-200 in lung cancer cells, modulating invasion, metastasis, and radiosensitivity.^19^^,^^28^ In this study, we showed that miR-200 directly targets NRP2, which interacts with VEGFR as a co-receptor for VEGF,^27^ and that miR-200 inhibits the ability of CAFs to promote the migration and tube formation of vascular endothelial cells. These results add to the existing evidence that miR-200 suppresses angiogenesis within the TME, further strengthening the argument that NRP2 is a promising target for anticancer therapeutics.^45^^,^^46^ Owing to the critical role of NRP2 in modulating CAF function, NRP2 blockade is a potential strategy for disrupting the interactions between CAFs, cancer cells, and other tumor-associated stromal cells within the TME.

Communication between CAFs and cancer cells occurs through direct physical contact or indirect paracrine signaling.^47^ The present findings, obtained with transwell systems and CMs, highlight the importance of indirect interactions between CAFs and cancer cells. Among the secretory factors downregulated in CAF-200 cells, VEGF-D and PTN demonstrated the ability to enhance cancer cell migration and invasion. VEGF-D has been implicated in promoting tumor growth, lymphangiogenesis, and lymphatic metastasis in lung cancer.^32^^,^^48^ Similarly, PTN can stimulate the proliferation and migration of lung cancer cells,^31^^,^^49^ and serum PTN levels are associated with lung cancer progression.^50^ Intriguingly, PTN promotes cancer cell migration and invasion by directly binding to NRP1, a protein closely related to NRP2.^51^ Although our study identified the roles of PTN and VEGF-D in the interplay between CAFs and cancer cells, further investigation is warranted to explore the crosstalk between NRP2 and these proteins.

Although this study provides valuable insights into the role of miR-200 in regulating CAF activity within the TME, several limitations need to be acknowledged. First, the mechanisms underlying the downregulation of miR-200 expression in CAFs are not fully understood. Transcription factors such as ZEB1^35^ and GATA3^22^ and promoter methylation^52^ can regulate miR-200 expression in a CAF-specific manner. Second, because miR-200 is transferred via extracellular vesicles,^53^ exosomal miR-200 may participate in the interaction between CAFs and cancer cells. However, in our preliminary study, GW4869, an inhibitor of exosome generation, had a minimal effect on CAF activity, implying that exosomal miR-200 has only a minor effect on CAF activation. Additionally, the precise mechanisms by which PTN and VEGF-D originating from CAFs contribute to cancer cell behavior have not been fully elucidated.

In conclusion, miR-200 inhibits the activation of CAFs by targeting NRP2/VEGFR signaling, preventing CAFs from promoting cancer cell motility and invasiveness, reconstructing the ECM, recruiting macrophages and vascular endothelial cells, and facilitating angiogenesis. Determining the clinical relevance of these findings will enhance the prospects of miR-200 and NRP2 as potential therapeutic targets in the treatment of lung cancer.

## Materials and methods

### Cell culture

Lung cancer cell lines 344SQ, 344LN, 531LN2 (murine), and A549 (human) were cultured in RPMI 1640 medium (Welgene, Gyeongsan, Korea) with 10% fetal bovine serum (FBS; HyClone, Logan, UT, USA) at 37°C in the presence of 5% CO_2_. A549 cells were authenticated by short tandem repeat profiling using the Powerplex 18D kit (Promega, Madison, WI, USA). Lung CAFs and LFs were isolated as previously described^2^^,^^19^ and maintained in alpha-MEM (Welgene) containing 10% FBS, penicillin/streptomycin (100 U/mL and 100 μg/mL; Welgene), 2 mM L-glutamine (Welgene), and 1 mM sodium pyruvate (Welgene). Raw264.7 and IMAECs were cultured in DMEM (Welgene) with 10% FBS. Lung cancer cells and fibroblasts were stably labeled with mCherry and GFP, as described previously,^2^ or transiently labeled with CellTracker (Invitrogen, Waltham, MA, USA). For miR-200 overexpression, the miR-200b/200a/429 cluster in the pLenti4.1 vector^20^ was subcloned into the pLVX-Hyg vector, which was modified from pLVX-Puro (Clontech, Mountain View, CA, USA). miR-200_pLVX-Hyg was introduced into LFs via lentiviral infection. miR-200b mimic, miR-200b inhibitor, and siRNAs (against *Nrp2*, *Vegfd*, *Ptn*, *Flt1*, and *Kdr*) were purchased from Bioneer (Daejeon, Korea) and transiently transfected into fibroblasts using the TransIT-X2 Dynamic Delivery System (Mirus Bio, Madison, WI, USA). The siRNA sequences used in this study are listed in Table S1. Human *NRP2* cDNA (pCNS-D2_NRP2, #KU027145) was purchased from the Korean Human Gene Bank, Medical Genomics Research Center, KRIBB, Korea, and subcloned into the pcDNA3.1 vector (Invitrogen). Murine *Nrp2* shRNAs were cloned into the pLKO.1 vector (a gift from David Root, Addgene plasmid #10878). For actin staining, fibroblasts were stained with Alexa Fluor 594 Phalloidin (Invitrogen) according to the manufacturer’s protocol. Cell viability was measured using the Quanti-MAX WST-8 Cell Viability Assay Kit (Biomax, Guri, Korea). The fibroblasts (5 × 10^5^ cells) were cultured in a 90-mm dish with 5 mL serum-free alpha-MEM for 24 h to obtain a CM. The CM was then filtered through a 0.45-μm syringe filter and stored at −80°C until required. NRP2 neutralizing antibody (#AF2215; R&D Systems, Minneapolis, MN, USA), VEGF-A (#CYT-336; ProSpec, Rehovot, Israel), VEGF-D (#PKSM041181; Elabscience, Houston, TX, USA), and PTN (#PKSM041292; Elabscience) recombinant proteins were purchased.

### RT-qPCR

Total RNA was isolated from the cells using the XENOPURE Total RNA Purification Kit (Xenohelix, Incheon, Korea) according to the manufacturer’s protocol. To analyze mRNA levels, RT-qPCR assays were performed using a BioFACT A-Star Real-time PCR Kit with SFCgreen I (BioFACT, Daejeon, Korea) after reverse transcription with TOPscript RT DryMIX (Enzynomics, Daejeon, Korea). The mRNA levels were normalized to *RPL32* mRNA. The sequences of the RT-qPCR primers used in this study are presented in Table S2. The miR levels were quantified using an HB miR Multi Assay Kit (Heimbiotek, Seongnam, Korea) according to the manufacturer’s protocol and normalized to the levels of *RNU6B* snoRNA. RNA isolation from FFPE tumor samples and RT-qPCR were performed as previously described.^2^ All experiments were performed at least three times, and the representative results are presented herein.

### Immunocytochemistry

CAFs were seeded on collagen-coated coverslips and cultured for 24 h. The cells were then fixed with 3.7% formaldehyde and subjected to staining with anti-α-SMA antibody (1:100 dilution, #14-9760-82; Invitrogen) and Alexa Fluor 594-conjugated anti-mouse antibody (1:200 dilution, Invitrogen). DAPI staining was performed to visualize the nuclei, and the slides were observed through fluorescence microscopy (Leica DMi8; Leica, Wetzlar, Germany).

### Collagen gel contraction assay

Lung cancer cells (5 × 10^4^ cells/well) and fibroblasts (1 × 10^5^ cells/well) were mixed with collagen (1 mg/mL) and co-cultured in a 24-well plate. After incubation for 24 h, the size of the collagen gel was measured using the ImageJ software (http://imagej.nih.gov/ij).

### Spheroid invasion assay

For spheroid formation, a mixture of lung cancer cells (5 × 10^4^ cells) labeled with red fluorescence and GFP-labeled fibroblasts (1 × 10^5^ cells) in 5 mL complete medium containing 20% METHOCEL (Sigma-Aldrich, St. Louis, MS, USA) and 1% Matrigel (BD Biosciences, Franklin Lakes, NJ, USA) was suspended on the lids of 150-mm dishes and incubated at 37°C for 2 days. Subsequently, the spheroid mixture (consisting of spheroids in 0.5× PBS, 0.01 N NaOH, and 3 mg/mL collagen) was implanted at the center of each well in a 12-well plate. Once the gels were polymerized, the wells were filled with the cell culture medium. After 1–2 days, images of the invading cells were captured using phase-contrast and fluorescence microscopy, and the invasion ratio was calculated by dividing the total invaded area by the central spheroid area using the ImageJ software for measurement.

### Transwell migration assay

Fibroblasts (1 × 10^5^ cells/well) were seeded into 24-well plates a day prior to the experiment. Subsequently, lung cancer cells (1 × 10^5^ cells/well) were seeded in hanging inserts (Labselect, Hefei, China) in serum-free medium. The cells were then incubated at 37°C for 24 h, and the migrated cancer cells were stained with 0.1% crystal violet. A mixture of red-labeled cancer cells (5 × 10^4^ cells per well) and green-labeled fibroblasts (5 × 10^4^ cells per well) was seeded in hanging inserts, and migrated cells were observed through fluorescence microscopy. For quantification, three randomly selected microscopic fields (×100 magnification) per chamber were captured and the migrated cells were counted manually using the ImageJ Cell Counter plug-in.

### Two-well migration assay

In separate cultures, mCherry-labeled 344SQ cells (3.5 × 10^4^ cells per well) and GFP-labeled CAFs (1.4 × 10^4^ cells per well) were cultured using the Culture-Insert 2 Well in μ-Dish 35 mm (Ibidi, Gräfelfing, Germany), which included a 500-μm wall to separate the cells. Following a 24-h incubation, the wall was removed to allow the cells to migrate toward each other. After an additional 24 h, the overlapping region between the two cell types was captured through fluorescence microscopy and measured using ImageJ software.

### Mouse experiments

The proposed mouse studies were approved by the Institutional Animal Care and Use Committee (IACUC) of Ewha Womans University College of Medicine (EWHA MEDIACUC past-060-5) prior to initiation. The mice were euthanized in accordance with the IACUC guidelines. Syngeneic (129/Sv) mice were subcutaneously injected into the right flank or orthotopically injected in the left lung with 344SQ (5 × 10^5^ cells per mouse) and CAFs (5 × 10^5^ cells per mouse). Syngeneic mice were also injected via the lateral tail veins with 344SQ (2 × 10^5^ cells) and CAFs (1 × 10^5^ cells). The mice injected subcutaneously were euthanized after 6 weeks, and those injected orthotopically or intravenously were euthanized after 1 week, and the primary and metastatic tumors were analyzed.

### RNA sequencing

Total RNAs were extracted in triplicate from vector-transduced CAFs (CAF-vec) and CAF-200 cells using the XENOPURE Total RNA Purification Kit and subsequently sent to Clinomics (Ulsan, Korea) for RNA sequencing. The quantity and integrity of the total RNA were assessed, followed by the construction of an RNA library using the Illumina TruSeq Stranded mRNA Sample Prep kit (Illumina, San Diego, CA, USA) from 1 μg total RNA. Subsequently, paired-end (2 × 100 bp) sequencing of the indexed library was performed using Illumina NovaSeq (Illumina). Differential gene expression was analyzed using edgeR, and enrichment analysis of genes downregulated in CAF-200 was performed using Metascape.^54^

### Western blot

Cell lysates were prepared using a lysis buffer (50 mM Tris-Cl pH 7.4, 150 mM NaCl, 1 mM EDTA, and 1% Triton X-100) supplemented with protease inhibitors (Sigma-Aldrich). Subsequently, the proteins (50 μg) were separated by SDS-PAGE, transferred onto PVDF membranes (Bio-Rad; Hercules, CA, USA), and incubated with primary antibodies and horseradish peroxidase-conjugated secondary antibodies (Bio-Rad). Protein bands were visualized using the Miracle-Star Western Blot Detection System (iNtRON Biotechnology, Seongnam, Korea). Antibodies against NRP2 (1:1,000 dilution, #sc-13117; Santa Cruz Biotechnology, Dallas, TX, USA), phospho-FAK (1:1,000 dilution, #sc-81493; Santa Cruz Biotechnology), FAK (1:1,000 dilution, #sc-557; Santa Cruz Biotechnology), phospho-MAPKAPK2 (1:1,000 dilution, #3041; Cell Signaling Technology, Danvers, MA, USA), MAPKAPK2 (1:1,000 dilution, #3042; Cell Signaling Technology), and β-actin (1:5,000 dilution, #BS6007M; Bioworld Technology, St. Louis Park, MN, USA) were used.

### 3′-UTR reporter assay

A segment of the murine *Nrp2* 3′-UTR (1,224 bp) containing potential miR-200-binding sites was amplified from 344SQ genomic DNA through PCR and inserted into the pCI-neo-hRL vector. The putative miR-200-binding sites within the *Nrp2* 3′-UTR were identified using TargetScan. Subsequently, 3′-UTR reporters (500 ng) and pGL3-control (50 ng; Promega) were co-transfected into 293T cells seeded in 24-well plates (1 × 10^5^ cells/well) using the TransIT-X2 transfection reagent (Mirus Bio). The transfections were performed in the presence or absence of miR-200 mimic (50 nM; Bioneer). Two days after transfection, luciferase activity was measured using the Luc-Pair Duo-Luciferase Assay Kit (GeneCopoeia, Rockville, MD, USA).

### ELISA

VEGF-D and PTN levels in CM from CAF-vec and CAF-200 cells were measured using mouse VEGF-–D and PTN ELISA Kits (Biorbyt, Cambridge, UK) according to the manufacturer’s protocol.

### Flow cytometry

Raw264.7 cells treated with CM from CAF-vec and CAF-200 were stained with antibodies against F4/80 (conjugated with FITC, #123107; BioLegend, San Diego, CA, USA) and CD86 (conjugated with APC, #105011, BioLegend) and were subjected to flow cytometry (NovoCyte 3000; ACEA Biosciences, San Diego, CA, USA).

### Immunohistochemistry

FFPE slides were deparaffinized in xylene, rehydrated in ethanol, and then incubated with antibodies against F4/80 (#ab16911, Abcam, Cambridge, UK), CD206 (#ab64693, Abcam), and CD31 (#ab182981, Abcam) followed by incubation with Alexa Fluor 488– and Alexa Fluor 594–conjugated secondary antibodies (Invitrogen).

### Wound healing assay

Artificial scratches were created on confluent cell monolayers in six-well plates using a pipette tip. The wound area was subsequently measured at specified time points using ImageJ software.

### Tube formation assay

A suspension containing 5 × 10^3^ IMAEC was prepared in 100 μL serum-free DMEM and plated on solidified Matrigel in a 96-well plate. Subsequently, 100 μL of either DMEM with 20% FBS or CM from CAF-vec and CAF-200 was added to the respective wells. Following a 4-h incubation period, the number of fully formed tube networks within five randomly chosen microscopic fields was determined.

### Statistical analysis

Data were analyzed using Student’s *t* test, Spearman’s rank correlation test, ANOVA, and log rank test using GraphPad Prism (La Jolla, CA, USA) unless otherwise noted. In Kaplan-Meier analyses, the optimal cutoff with the most significant (log rank test) split was determined using the survminer R package. *p* Values of <0.05 were considered statistically significant. The raw data for all graphs in the manuscript are presented in Table S3.

## Data and code availability

The RNA sequencing datasets used in this study are available in the NCBI Gene Expression Omnibus (GEO) database under accession number (GEO: GSE226886). The other datasets analyzed in this study are available from the corresponding author upon reasonable request.

## Supplemental information

Document S1. Figures S1–S7 and Tables S1–S3

Table S3. The raw data for all graphs in the manuscript

Document S2. Article plus supplemental information
