# Supplementary material for: miR-200-mediated inactivation of cancer-associated fibroblasts via targeting of NRP2-VEGFR signaling attenuates lung cancer invasion and metastasis
Source: Mol Ther Nucleic Acids. 2024 Apr 23;35(2):102194. doi: 10.1016/j.omtn.2024.102194 (PMC11101731; doi:10.1016/j.omtn.2024.102194)
Supplement: Document S1. Figures S1–S7 and Tables S1–S3 [file mmc1.pdf]

OMTN, Volume *35*

Supplemental information

miR-200-mediated inactivation of cancer-associated fibroblasts via targeting of NRP2-VEGFR signaling attenuates lung cancer invasion and metastasis

Inyoung Cheon, Sieun Lee, Seonyeong Oh, and Young-Ho Ahn

Supplemental Information

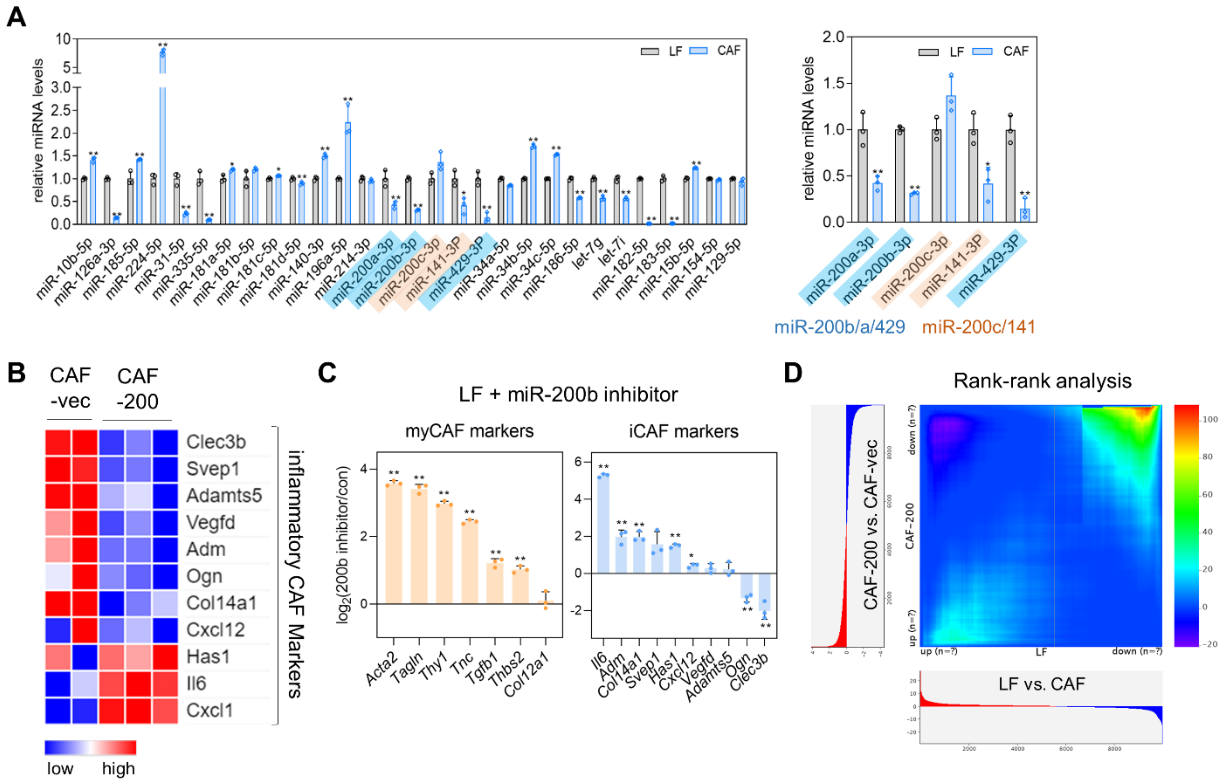

Figure S1. miR-200 downregulated in CAFs suppresses the expression of CAF markers.

A. RT-qPCR analysis of miRNAs differentially expressed between LFs and CAFs. miRNA levels were normalized to *RNU6B* snoRNA levels, and values relative to those of LFs (set at 1.0) are presented. The results of miR-200 family members are shown on the right. Mean + SD (n = 3). * $P < 0.05$, ** $P < 0.01$; two-tailed Student’s t-test.

B. A heatmap showing the expression of inflammatory CAF makers in RNA sequencing data from CAF-vec and CAF-200.

C. RT-qPCR analysis of myofibroblastic (myCAF) and inflammatory CAF (iCAF)-specific markers in LFs transfected with miR-200b inhibitor. The expression was normalized to the *Rpl32* mRNA level, and log2 fold-change values (miR-200 inhibitor vs. control) are presented in the graphs. Mean + SD (n = 3). *$P < 0.05$, **$P < 0.01$; two-tailed Student’s t-test.

D. Rank–rank hypergeometric overlap analysis of two profiling data sets: CAF-200 vs. CAF-vec and LFs vs. CAFs. Negative values of log10-transformed hypergeometric p-values are indicated in the color scale.

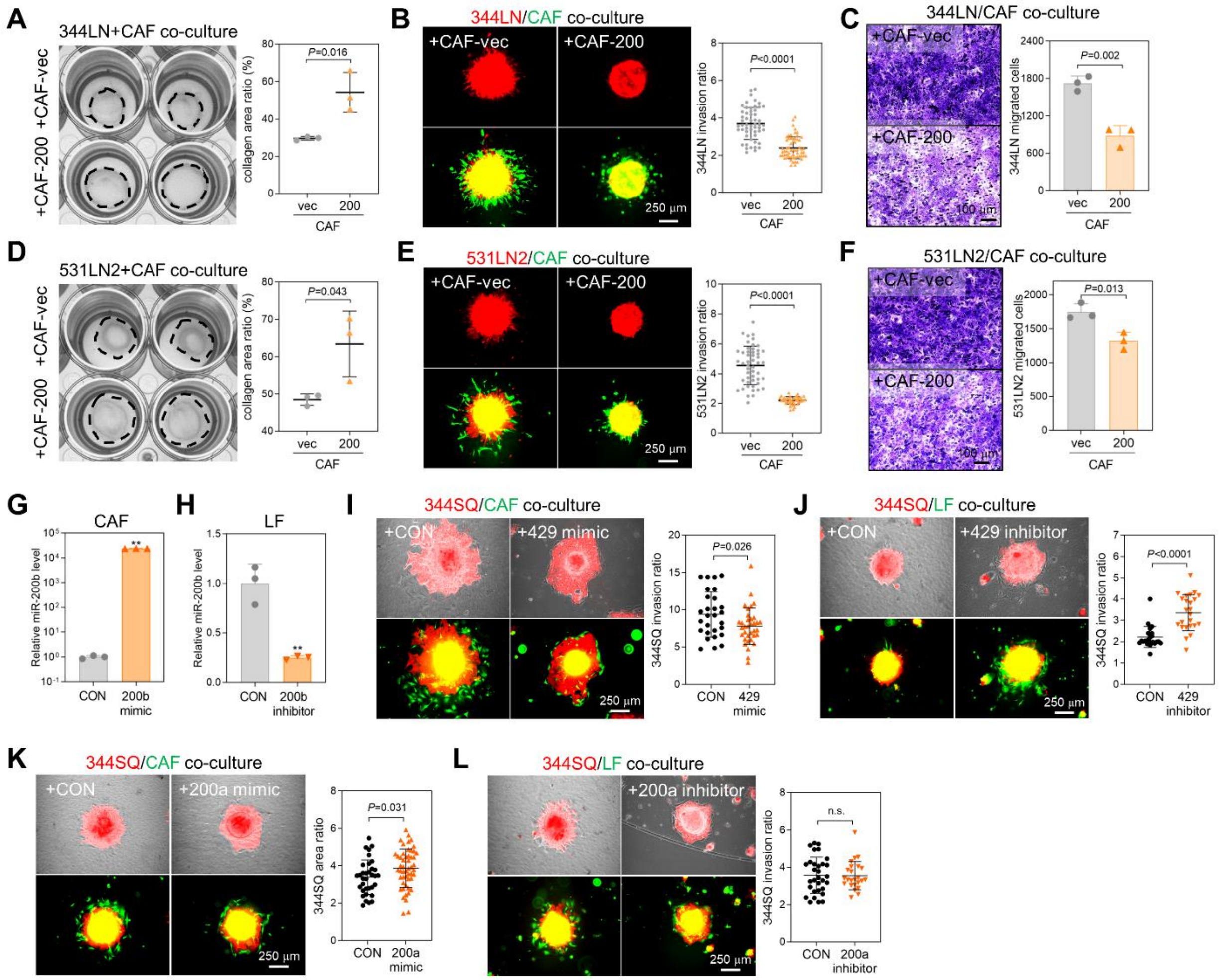

Figure S2. miR-200 prevents CAFs from facilitating cancer cell migration and invasion.

A, D. Collagen gel contraction assay of 344LN (A) and 531LN2 cells (D) co-cultured with CAF-vec and CAF-200. The collagen area was measured using ImageJ software. Mean ± SD (n = 3). *P*, two-tailed Student's *t*-test.

B, E. Spheroid invasion assay of 344LN (B) and 531LN2 cells (E) co-cultured with CAF-vec or CAF-200. 344LN and 531LN2 cells were labeled with mCherry (red fluorescence), and fibroblasts were labeled with GFP. Spheroids prepared from hanging-drop cultures were seeded on collagen gels and cultured for 2 days. Spheroid invasion ratios (ratio of whole cell area to central spheroid area) were measured using ImageJ software. Mean ± SD (for 344LN, +CAF-vec, n = 54; +CAF-200, n = 61; for 531LN2, +CAF-vec, n = 50; +CAF-200, n = 41). *P*, two-tailed Student's *t*-test.

C, F. Transwell migration assay of 344LN (C) and 531LN2 cells (F) co-cultured with CAF-vec or CAF-200. Fibroblasts were seeded in the bottom wells, and 344LN and 531LN2 cells were seeded in the upper wells. After 24 h, the migrated cancer cells were stained with crystal violet, photographed, and counted. Mean + SD (n = 3). *P*, two-tailed Student's *t*-test.

G. RT-qPCR analysis of *miR-200b* expression in human CAFs transfected with control (CON) or miR-200b mimic. *miR-200b* levels were normalized to *RNU6B* snoRNA levels, and values relative to those of the control (set at 1.0) are presented. Mean + SD (n = 3). ** $P < 0.01$, two-tailed Student's *t*-test.

H. RT-qPCR analysis of *miR-200b* expression in murine LFs transfected with control (CON) or miR-200b inhibitor. Mean + SD (n = 3). ** $P < 0.01$, two-tailed Student's *t*-test.

I. Spheroid invasion assay of 344SQ cells (labeled with mCherry) co-cultured with CAFs (labeled with CellTracker Green) transfected with the control (+CON) or miR-429 mimic (+429 mimic). Mean ± SD (+CON, n = 28; +429 mimic, n = 35). *P*, two-tailed Student's t-test.

J. Spheroid invasion assay of 344SQ cells co-cultured with LFs (labeled with CellTracker Green) transfected with the control (+CON) or miR-429 inhibitor (+429 inhibitor). Mean ± SD (+CON, n = 25; +429 inhibitor, n = 24). *P*, two-tailed Student's t-test.

K. Spheroid invasion assay of 344SQ cells co-cultured with CAFs transfected with the control (+CON) or miR-200a mimic (+200a mimic). Mean ± SD (+CON, n = 51; +200a mimic, n = 43). *P*, two-tailed Student's t-test.

L. Spheroid invasion assay of 344SQ cells co-cultured with LFs transfected with the control (+CON) or miR-200a inhibitor (+200a inhibitor). Mean ± SD (+CON, n = 31; +200a inhibitor, n = 24). n.s., not significant.

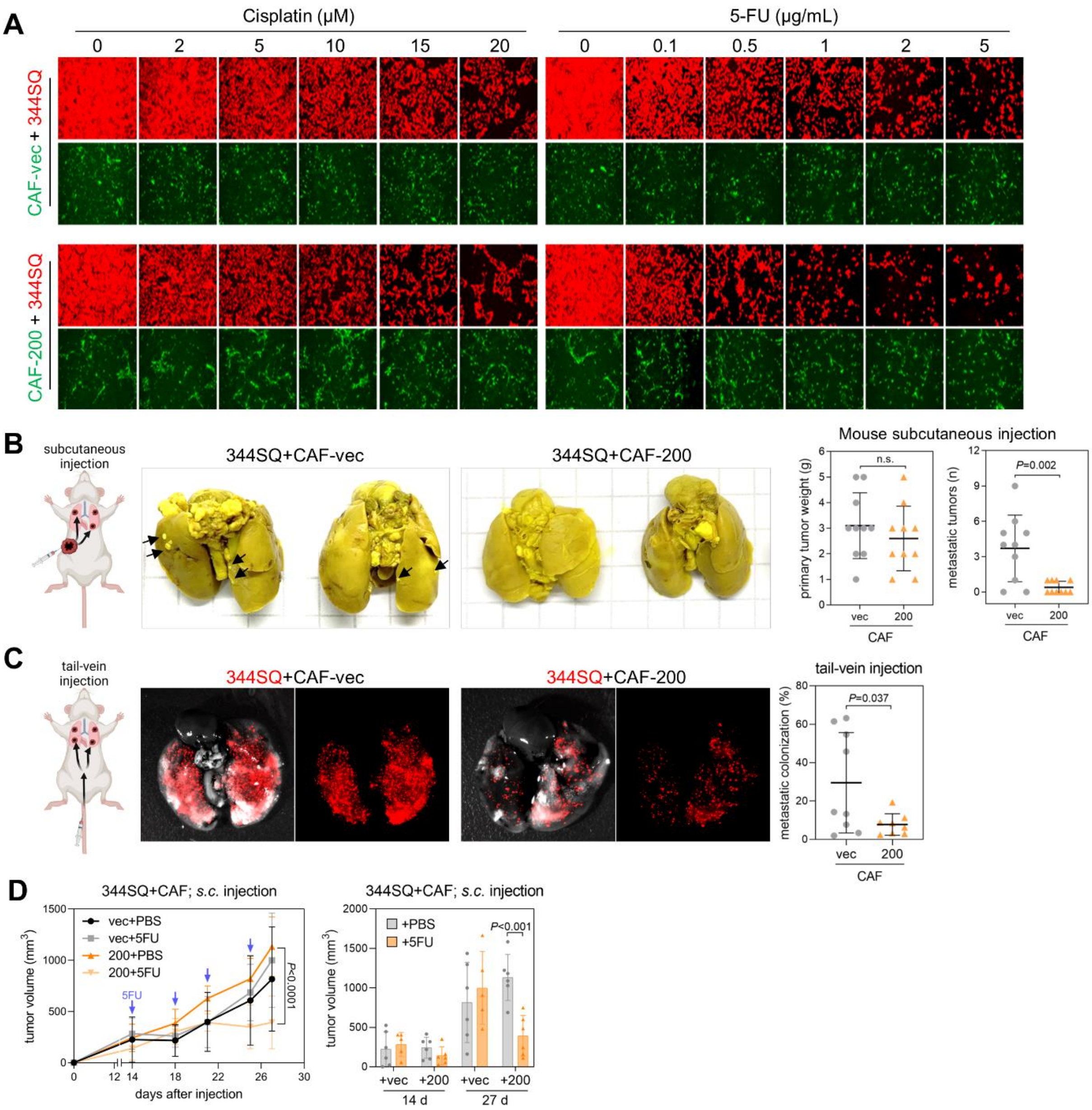

Figure S3. miR-200 prevents CAFs from facilitating the drug resistance of lung cancer cells.

A. Viability of 344SQ cells treated with cisplatin or 5-FU in the presence of CAF-vec or CAF-200. 344SQ cells (red fluorescence) were co-cultured with CAF-vec or CAF-200 (green fluorescence) and treated with cisplatin (0–20 µM) or 5-FU (0–5 µg/mL). After 48 h, the 344SQ cells and CAFs were photographed under a fluorescence microscope.

B. Subcutaneous injection of 344SQ cells (5×10^5 cells/mouse) with CAF-vec or CAF-200 (5×10^5 cells/mouse) into syngeneic mice (129/Sv). After 6 weeks, the mice were necropsied, tumors were weighed, and lungs were photographed. The graphs present the tumor weight and number of metastatic nodules in the lungs. Mean ± SD (n = 10). *P*, two-tailed Student's *t*-test.

C. Tail-vein injection of mCherry-labelled 344SQ cells (2×10^5 cells/mouse) with CAF-vec or CAF-200 (1×10^5 cells/mouse) into syngeneic mice (129/Sv). After a week, the mice were necropsied, lungs were photographed under a fluorescence stereomicroscope. The graph presents the relative area of metastatic nodules in the lungs. Mean ± SD (+CAF-vec, n = 9; +CAF-200, n = 8). *P*, two-tailed Student's *t*-test.

D. Drug resistance of lung cancer cells. 344SQ cells (5×10^5 cells/mouse) with CAF-vec or CAF-200 (5×10^5 cells/mouse) were subcutaneously injected into syngeneic mice. After two weeks, 5-FU (50 mg/kg) was injected intraperitoneally twice a week for another two weeks. Mean ± SD (n = 5~6). *P*, two-way ANOVA (left) or two-tailed Student's *t*-test (right).

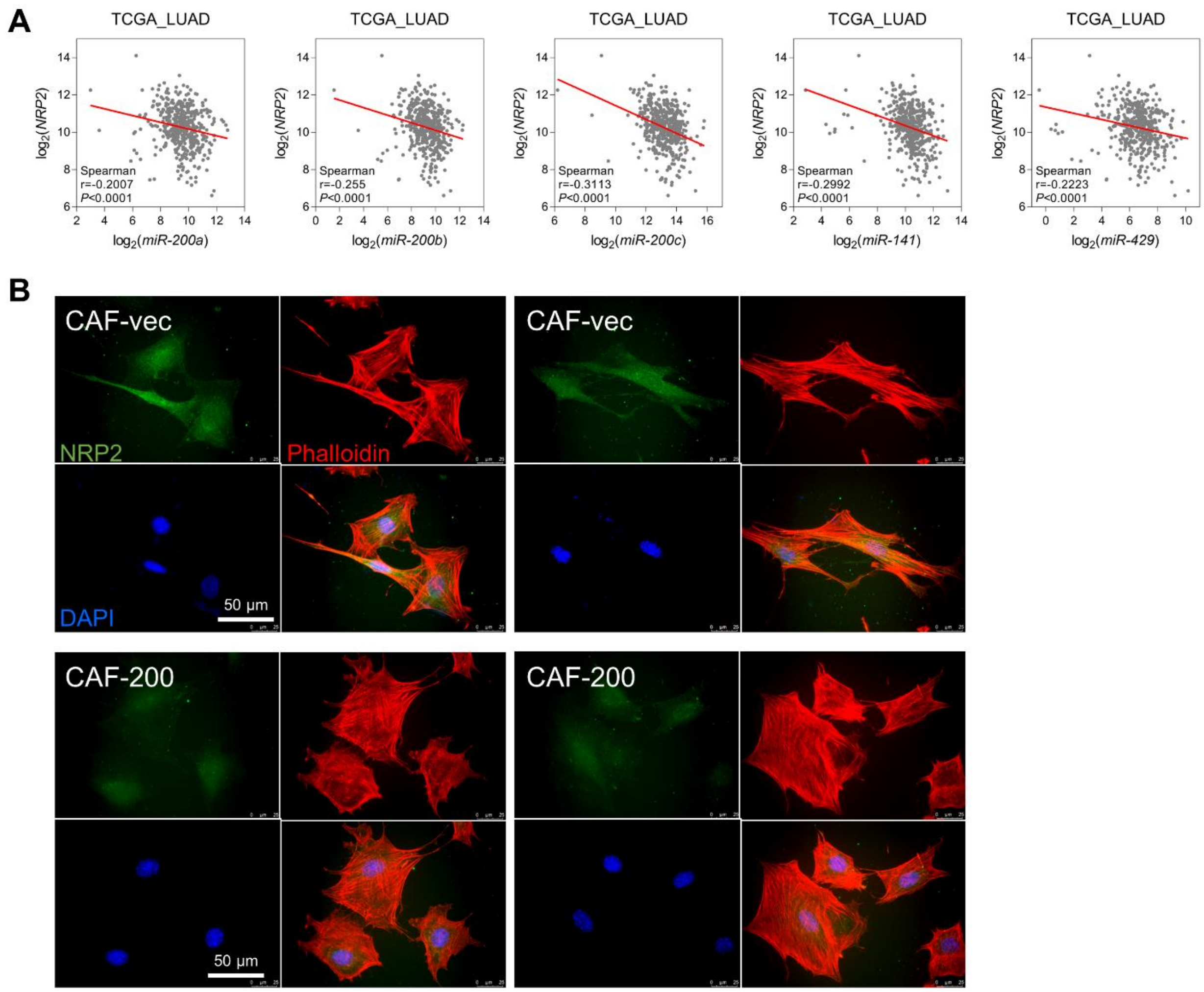

Figure S4. miR-200 levels are negatively correlated with *NRP2* levels.

A. Correlation between miR-200 family members (miR-200a, -200b, -200c, -141, and -429) and *NRP2* expression levels in TCGA-LUAD data. Spearman correlation coefficients (r) and *P*-values are shown in the graphs.

B. Immunocytochemistry of NRP2 in CAF-vec and CAF-200. CAFs were stained with NRP2 antibody (green), phalloidin (red), and DAPI (blue).

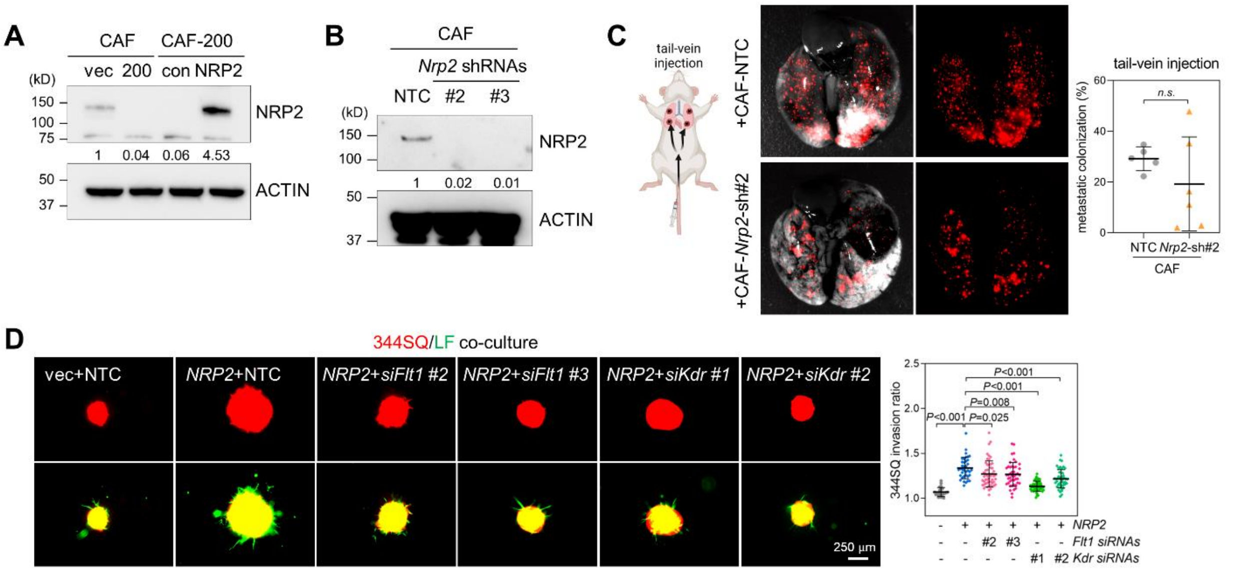

Figure S5. VEGFR1 and VEGFR2 are required for the NRP2-mediated regulation of CAF activation.

A. Western blot of NRP2 in CAF-200 transfected with an empty vector (+vec) or a NRP2-overexpressing vector (+NRP2). Actin was used as a control. Relative quantitation results are shown below.

B. Western blot of NRP2 in CAFs transfected with *Nrp2* shRNAs (#2 and #3) and non-targeting control (NTC). Actin was used as a control. Relative quantitation results are shown below.

C. Tail-vein injection of mCherry-labelled 344SQ cells (2×10^5 cells/mouse) with CAFs (1×10^5 cells/mouse) transfected with NTC or *Nrp2* shRNAs (#2 and #3) into syngeneic mice (129/Sv). After a week, the mice were necropsied, lungs were photographed under a fluorescence stereomicroscope. The graph presents the relative area of metastatic nodules in the lungs. Mean ± SD (+CAF-NTC, n = 5; +CAF-*Nrp2*-sh#2, n = 6).

D. Spheroid invasion assay of 344SQ cells co-cultured with LFs transfected with *NRP2* overexpressing vector and siRNAs against *Flt1* or *Kdr*. Mean ± SD (vec+NTC, n = 24; *NRP2*+NTC, n = 37; *NRP2*+si*Flt1* #2, n = 52; +si*Flt1* #3, n = 45; +si*Kdr* #1, n = 50; +si*Kdr* #2, n = 48). *P*, two-tailed Student's *t*-test.

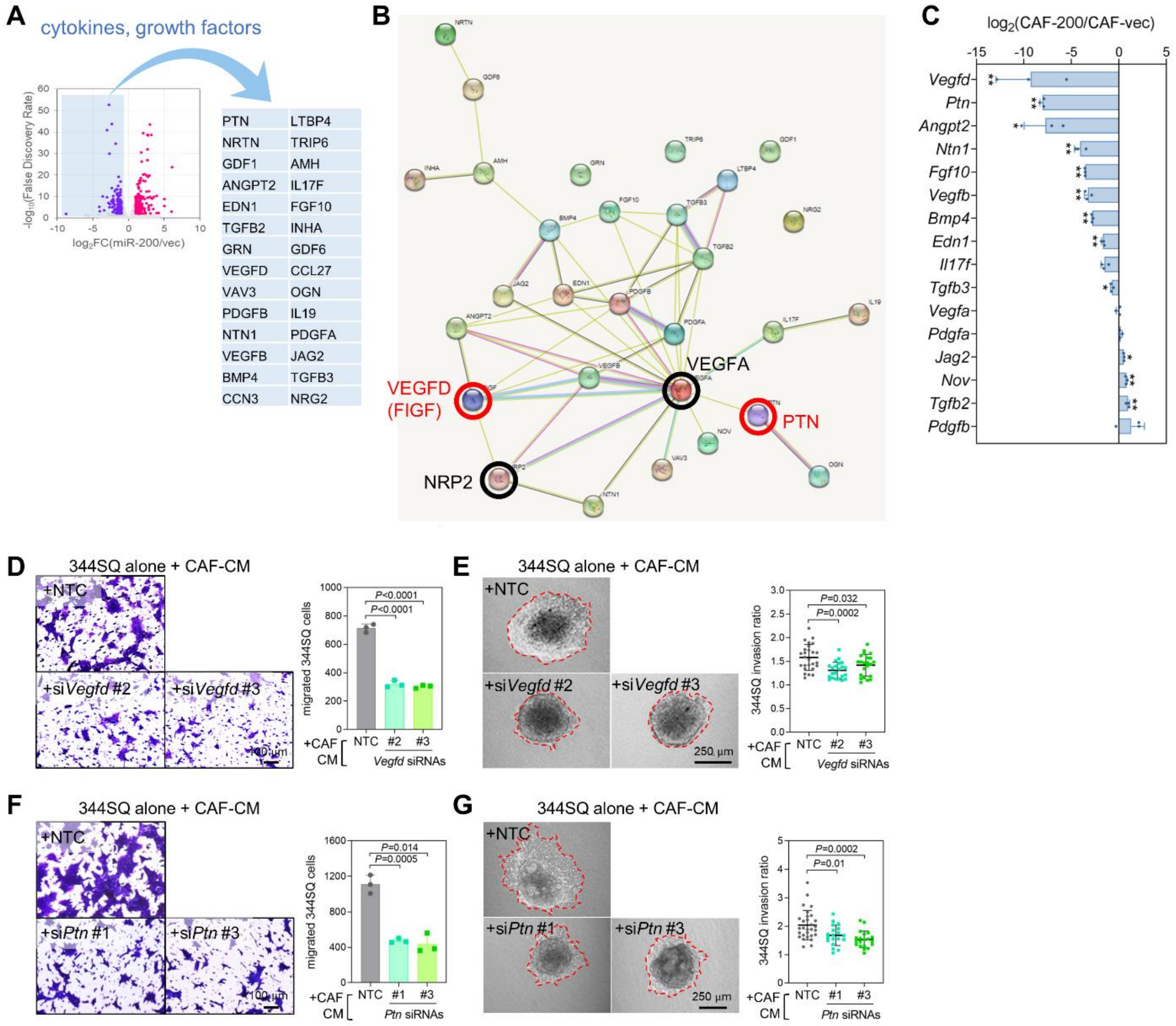

Figure S6. Cytokines and growth factors are downregulated by miR-200 in CAFs.

A. List of downregulated cytokines and growth factors in CAF-200 compared with those in CAF-vec based on the RNA sequencing data ($\log_2$ fold-change > 1, FDR < 0.01).

B. Functional protein association network using STRING database (https://string-db.org) between cytokines and growth factors downregulated by miR-200 in CAFs.

C. RT-qPCR analysis of cytokines ang growth factors in CAF-vec and CAF-200. The expression levels were normalized to the *Rpl32* mRNA level, and the expression ratios of CAF-200 to CAF-vec are presented. Mean + SD (n = 3). * $P < 0.05$, ** $P < 0.01$; two-tailed Student's *t*-test.

D. Transwell migration assay of 344SQ cells treated with CM from CAFs transfected with NTC or si*Vegfd*. 344SQ cells were seeded in the upper inserts, and CAF-CM were added to the bottom wells. After 24 h, the migrated 344SQ cells were photographed and counted. Mean + SD (n = 3). *P*, two-tailed Student's *t*-test.

E. Spheroid invasion assay of 344SQ cells with CM of CAFs transfected with NTC or si*Vegfd*. 344SQ spheroids were incubated with CAF-CM for 24 h. Mean ± SD (+NTC, n = 26; +si*Vegfd* #2, n = 22; +si*Vegfd* #3, n = 22). *P*, two-tailed Student's *t*-test.

F. Transwell migration assay of 344SQ cells with CM of CAFs transfected with NTC or si*Ptn*. Mean + SD (n = 3). *P*, two-tailed Student's *t*-test.

G. Spheroid invasion assay of 344SQ cells with CM of CAFs transfected with NTC or si*Ptn*. Mean ± SD (+NTC, n = 29; + si*Ptn* #1, n = 19; +si*Ptn* #3, n = 20). *P*, two-tailed Student's *t*-test.

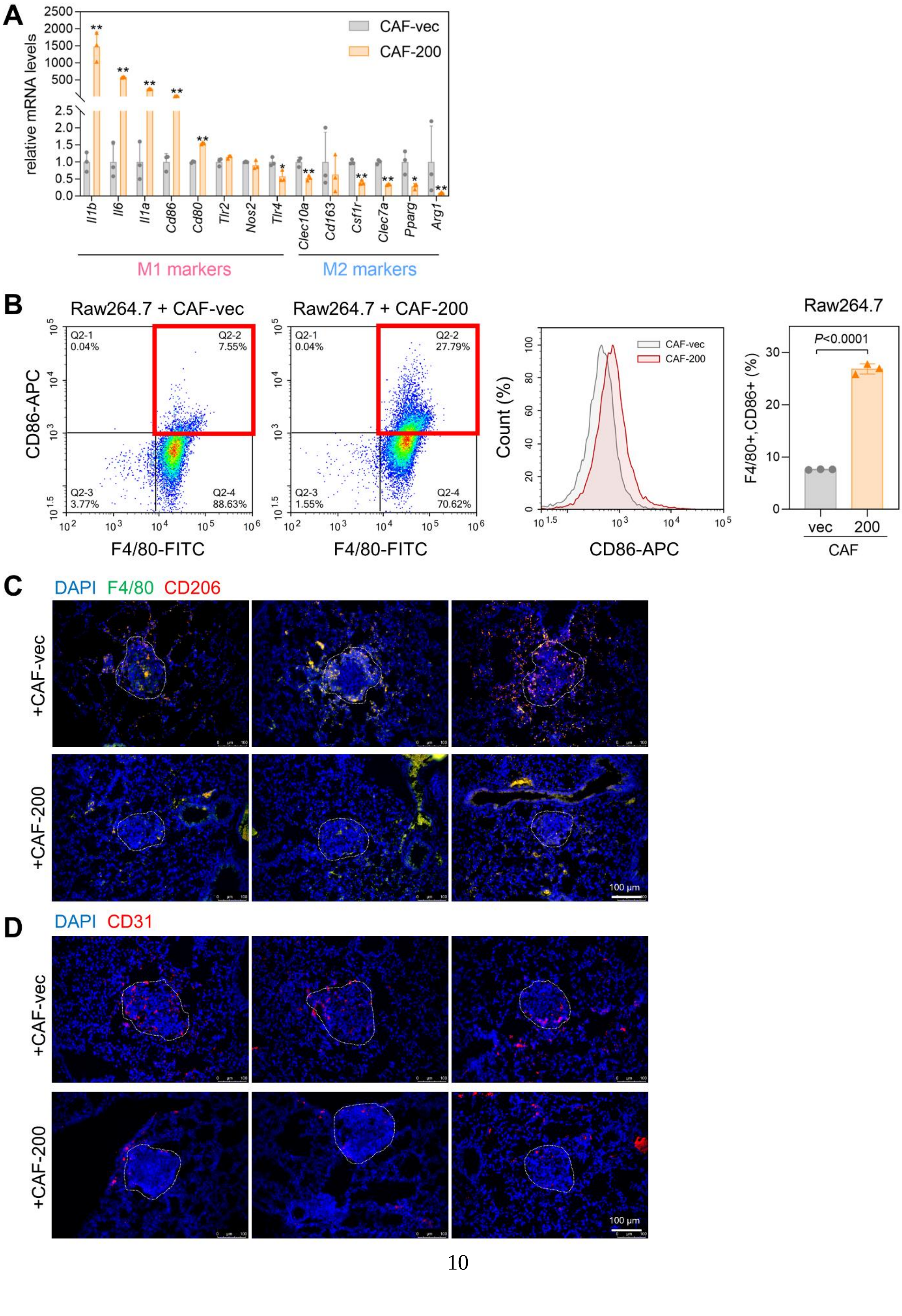
A
relative mRNA levels
CAF-vec
CAF-200
Il1b
Il6
Il1a
Cd86
Cd80
Tlr2
Nos2
Tlr4
Clec10a
Cd163
Csf1r
Clec7a
Pparg
Arg1
M1 markers
M2 markers
B
Raw264.7 + CAF-vec
Raw264.7 + CAF-200
CD86-APC
F4/80-FITC
Q2-1 0.04%
Q2-2 7.55%
Q2-3 3.77%
Q2-4 88.63%
Q2-1 0.04%
Q2-2 27.79%
Q2-3 1.55%
Q2-4 70.62%
Count (%)
CAF-vec
CAF-200
CD86-APC
Raw264.7
P<0.0001
F4/80+,CD86+ (%)
vec
200
CAF
C
DAPI F4/80 CD206
+CAF-vec
+CAF-200
100 μm
D
DAPI CD31
+CAF-vec
+CAF-200
100 μm

Figure S7. miR-200 attenuates CAF activity to recruit macrophages and vascular endothelial cells.

A. RT-qPCR analysis of M1 and M2 macrophage markers in Raw264.7 cells treated with CM of CAF-vec and CAF-200. The expression was normalized to the *Rpl32* mRNA level, and values relative to those of CAF-vec (set at 1.0) are presented. Mean + SD (n = 3). $*P < 0.05$, $**P < 0.01$; two-tailed Student's t-test.

B. Flow cytometry of F4/80 (a macrophage marker) and CD86 (an M1 marker) in Raw264.7 cells treated with CM of CAF-vec and CAF-200. The relative population of F4/80+ and CD86+ cells was shown in the graph. Mean + SD (n = 3). *P*, two-tailed Student's *t*-test.

C, D. Immunohistochemistry of F4/80, CD206 (an M2 marker; C), and CD31 (an endothelial cell marker; D) on the lungs of mice injected with 344SQ cells in combination with either CAF-vec or CAF-200. Tumor areas are delineated by white dotted lines.

Table S1. List of siRNAs used in this study

gene	sense (5′ → 3′)	antisense (5′ → 3′)
Nrp2 #1	CCUCACUUUGAAAUCGAGAAA	UUUCUCGAUUUCAAAGUGAGG
Nrp2 #2	CCGUGAAGAGUGAAGAGACUA	UAGUCUCUUCACUCUUCACGG
Nrp2 #3	CCAGAGAAGUAUCCACACAAU	AUUGUGUGGAUACUUCUCUGG
Vegfd #1	UCAUUCCAAGAAACUCUGU	ACAGAGUUUCUUGGAAUGA
Vegfd #2	CCUCCUGAUUAUUUUUGCA	UGCAAAAAUAAUCAGGAGG
Vegfd #3	CUGCUUAGUCAUCGGUAGA	UCUACCGAUGACUAAGCAG
Ptn #1	CUAAGAACAAACGGUCUUA	UAAGACCGUUUGUUCUUAG
Ptn #2	CUUAUAACCCAUGGUCUUU	AAAGACCAUGGGUUAUAAG
Ptn #3	GUGUGUGUUUAAAGCUGUA	UACAGCUUUAAACACACAC
Flt #1	CUGUAGAUACCUCCCUACA	UGUAGGGAGGUAUCUACAG
Flt #2	CUCUACGACCUUAGACUGU	ACAGUCUAAGGUCGUAGAG
Flt #3	GUGAGUACAAAGCUCUGAU	AUCAGAGCUUUGUACUCAC
Kdr #1	CUCCUAAUGAGAGUUCCUU	AAGGAACUCUCAUUAGGAG
Kdr #2	GAGAACCUCACGUGGUACA	UGUACCACGUGAGGUUCUC
Kdr #3	CAGUAUCUUCUGCAAAACA	UGUUUUGCAGAAGAUACUG

Table S2. List of RT-qPCR primers used in this study

gene (human)	forward (5′ → 3′)	reverse (5′ → 3′)
RPL32	ACAAAGCACATGCTGCCCAGTG	TTCCACGATGGCTTTGCGGTTC
NRP2	GTCTCCTACAGCCTAAACGGCA	GGGTCAAACCTTCGGATGTCAG
**gene (mouse)**	**forward (5′ → 3′)**	**reverse (5′ → 3′)**
Rpl32	ATCAGGCACCAGTCAGACCGAT	GTTGCTCCCATAACCGATGTTGG
Zeb1	ATTCAGCTACTGTGAGCCCTGC	CATTCTGGTCCTCCACAGTGGA
Pdgfra	GCAGTTGCCTTACGACTCCAGA	GGTTTGAGCATCTTCACAGCCAC
Fap	CCGCGTAACACAGGATTCACTG	CACACTTCTTGCTCGGAGGAGA
S100a4	AGCTCAAGGAGCTACTGACCAG	GCTGTCCAAGTTGCTCATCACC
Col1a2	TTCTGTGGGTCCTGCTGGGAAA	TTGTCACCTCGGATGCCTTGAG
Vim	GCGTGCGGCTGCTTCAAGAC	ATGGCGTCGGCCAGCGAGAA
Des	GCGGCTAAGAACATCTCTGAGG	ATCTCGCAGGTGTAGGACTGGA
Ddr2	TCATCCTGTGGAGGCAGTTCTG	CTGTTCACTTGGTGATGAGGAGC
P4ha3	TGCAAGTGGAGTACCGCATCAG	CACCTGGAGATACTCTGCGTAG
Itga1	GGCAGTGGCAAGACCATAAGGA	CATCTCTCCGTGGATAGACTGG
Acta2	TGCTGACAGAGGCACCACTGAA	CAGTTGTACGTCCAGAGGCATAG
Tagln	GCAGATGGAACAGGTGGCTCAA	CCCAAAGCCATTAGAGTCCTCTG
Thy1	CCTTACCCTAGCCAACTTCACC	TTATGCCGCCACACTTGACCAG
Tnc	GAGACCTGACACGGAGTATGAG	CTCCAAGGTGATGCTGTTGTCTG
Tgfb1	TGATACGCCTGAGTGGCTGTCT	CACAAGAGCAGTGAGCGCTGAA
Thbs2	GTATGGAGGGAAGGACTGTGTC	ACTTGGCTCCAGGAAAACACGG
Col12a1	CAGCACCATGAATGTCGTCTGG	GGTCTTTGAGGATAGTGCTGGC
Il6	TACCACTTCACAAGTCGGAGGC	CTGCAAGTGCATCATCGTTGTTC
Adm	GCCAGATACTCCTTCGCAGTTC	AGGAACTGTCGTCTCATCAGCG
Col14a1	GTCAGGCTTCAGTGATGCTCTG	ATTTGCCACCGAGCACACAAGC
Svep1	CCAGATGGTTGGGAATCCTGTG	GGCTGAGTAGAAGCCATTCTCC
Has1	GCTACTTCCACTGTGTGTCCTG	CTAAGCATTCGGTTGGTGAGGTG

Cxcl12	GGAGGATAGATGTGCTCTGGAAC	AGTGAGGATGGAGACCGTGGTG
Vegfd	CTCCACCAGATTTGCGGCAACT	ACTGGCGACTTCTACGCATGTC
Adamts5	CTGCCTTCAAGGCAAATGTGTGG	CAATGGCGGTAGGCAAACTGCA
Ogn	AACGACCTGGAATCTGTGCCTC	TCGCTCCCGAATGTAACGAGTG
Clec3b	CGAGGAACTCAAGAACAGGATGG	GCCTCATGGAAGGTCTTCGGTT
Nrp2	GGTGAAGATTGGATGGTCTACCG	TGAACCGAGTCAGCAGTGGCAT
Pcnx	GAGCACAGTGTTCACGGCATTG	TTCCTCACCTGAGGCAGCACAT
Hebp1	ACAGACAAGCCAGTGGATGAGG	GGGCAAAAGAGACAGGGACTGT
Man2a1	GCAAAGTTCACAAGATTCTCTGCC	GATGGACACCACTGAATGCCGT
Edn1	CTACTTCTGCCACCTGGACATC	CGCACTGACATCTAACTGCCTG
Gas1	ACTGCGGCAAGCTTTTCAACGG	CTCTTTGACCGATTCGCAGATGG
Gpnmb	GGCTACTTCAGAGCCACCATCA	CTTTGCAGGTCACAGTGAAGTCC
Gata3	CAAGAGCAGCTCCTTCAACC	AGGGATGACATGTGTCTGGAG
Il1rl1	GGATTGAGGTTGCTCTGTTCTGG	TCGGGCAGAGTGTGGTGAACAA
Tpp1	GCGATACAACCTGACAGCCAAAG	AAACTGCCACCGAATAGGCGCA
Klf6	GGAAGGTTGTGAGTGGCGTTTTG	AGGTGGTCAGACCTGGAGAAAC
Lox	CATCGGACTTCTTACCAAGCCG	GGCATCAAGCAGGTCATAGTGG
Gpc6	AGAGGTTGCCAACCGAGTTTCC	TGCAAGGTCTCACAGTGGGCAA
Mn1	CAAGTCCCAGAACCCCAACAAC	GTCACCATCTGTGCAGTGGACA
Stx2	ATCAGGACGAGAATGGGAACCG	CGGAACAGGATCTGCGCTTCAT
Klf4	CTATGCAGGCTGTGGCAAAACC	TTGCGGTAGTGCCTGGTCAGTT
Abca1	GGAGCCTTTGTGGAACTCTTCC	CGCTCTCTTCAGCCACTTTGAG
Cyp26b1	TGAGAGCAGCAAGGAACATGGC	TCAAGGATGTGCTTGCACTGGC
Gaa	ACCGTCCAACTTCGTTAGAGGC	ATTGGTGGCTGGAGGCACAGAT
Amotl2	CAGAGGGACAATGAGCGATTGC	TCACGCTTGGAAGAGGTCCTCA
Nub1	GACGCATTCAGAGGACCAAACG	TGAGTGACCTGCCTGTCTGGTT
Arhgef10	CGTCATCGAGACGGTTTACAACG	TACCTCTGGCTCATCACGGCAT
Svil	GTTTGCTTCTGGAGAGGTCAAGG	CTGCTTCCGAATCAGCCTGTTC
Plagl1	TCAAGTGCTCGAAGGCTGAGTG	CACTGGTGAATCTTCTGTGGCG
Pcdhgc3	CAGAACACGCTGCGAAGTTGTG	GGCTTGAGAGAAACGCCAGTCA
Ostm1	TGTCCGTGGTTGCTGTGTCTGT	GGCAAAACTGGTGCTCGACTTG
Fam43a	TACGCTGTGAGCCTGCACTACT	CTGGTGATGTGCAGCTTCTTGC
Rnd3	GACCAGAGACTCTGGACAGTGT	GTGCTGACATCTGTCCGCAGAT
Naga	GCCGTCCCATTGCCTTTTCTTG	AGTCCAGGATGGACAGCACACT
Slc38a2	GCGTTGGCATTCAATAGCACCG	TCGTAGATGGGAAGAACAGCGG
Nacc2	AGCCTCATCAGCCAGATTGGGT	TGCACACAGGTGGCAGTTCATG
Adamts1	GAAGGCAAACGAGTCCGCTACA	TTGGGTGTCCACTCTACAGTGG
Atp6v1b2	TGGCTCCAAAGCAGTGGTTCAG	CTCAGACACTGGTGTTCGGAGA
Tgfb2	TTGTTGCCCTCCTACAGACTGG	GTAAAGAGGGCGAAGGCAGCAA
Plin3	GATGTGGCTGAGAAAGGCGTCA	AGGCTTTCCTGCAGTCTGTCCA
Ifit3	GCTCAGGCTTACGTTGACAAGG	CTTTAGGCGTGTCCATCCTTCC
Dusp3	GCCACAGATTTCATTGACCAGGC	CGTCCATCTTCTGCCGCATCAT
Flt1	TGGATGAGCAGTGTGAACGGCT	GCCAAATGCAGAGGCTTGAACG
Kdr	CGAGACCATTGAAGTGACTTGCC	TTCCTCACCCTGCGGATAGTCA
Flt4	AGACTGGAAGGAGGTGACCACT	CTGACACATTGGCATCCTGGATC
Ptn	CCCTTGCAACTGGAAGAAGCAG	GACAGTCAGCATTGTGCAGAGC
Angpt2	AACTCGCTCCTTCAGAAGCAGC	TTCCGCACAGTCTCTGAAGGTG
Ntn1	GTCTGGTGTGTGACTGTAGGCA	CCGAGCATGGAGGTTGCAGTTG
Fgf10	ATCACCTCCAAGGAGATGTCCG	CGGCAACAACTCCGATTTCCAC
Vegfb	ACTGGGCAACACCAAGTCCGAA	CACATTGGCTGTGTTCTTCCAGG
Bmp4	GCCGAGCCAACACTGTGAGGA	GATGCTGCTGAGGTTGAAGAGG
Edn1	CTACTTCTGCCACCTGGACATC	CGCACTGACATCTAACTGCCTG

Il17f	AACCAGGGCATTTCTGTCCCAC	GGCATTGATGCAGCCTGAGTGT
Tgfb3	AAGCAGCGCTACATAGGTGGCA	GGCTGAAAGGTGTGACATGGAC
Vegfa	CTGCTGTAACGATGAAGCCCTG	GCTGTAGGAAGCTCATCTCTCC
Pdgfa	CTGGCTCGAAGTCAGATCCACA	GACTTGTCTCCAAGGCATCCTC
Jag2	CGCTGCTATGACCTGGTCAATG	TGTAGGCGTCACACTGGAACTC
Nov	TGCGAAGCTGACCTGGAGGAAA	CCGCAGAACTTAGCCCTGTATG
Pdgfb	AATGCTGAGCGACCACTCCATC	TCGGGTCATGTTCAAGTCCAGC
Il1b	TGGACCTTCCAGGATGAGGACA	GTTCATCTCGGAGCCTGTAGTG
Il1a	ACGGCTGAGTTTCAGTGAGACC	CACTCTGGTAGGTGTAAGGTGC
Cd86	ACGTATTGGAAGGAGATTACAGCT	TCTGTCAGCGTTACTATCCCGC
Cd80	CCTCAAGTTTCCATGTCCAAGGC	GAGGAGAGTTGTAACGGCAAGG
Tlr2	ACAGCAAGGTCTTCCTGGTTCC	GCTCCCTTACAGGCTGAGTTCT
Nos2	GAGACAGGGAAGTCTGAAGCAC	CCAGCAGTAGTTGCTCCTCTTC
Tlr4	AGCTTCTCCAATTTTTCAGAACTTC	TGAGAGGTGGTGTAAGCCATGC
Clec10a	ATGGGTGGATGGGACCGACTTT	GGAAGGTTCTCTGGCAGACATC
Cd163	GGCTAGACGAAGTCATCTGCAC	CTTCGTTGGTCAGCCTCAGAGA
Csf1r	TGGATGCCTGTGAATGGCTCTG	GTGGGTGTCATTCCAAACCTGC
Clec7a	CCAGCTAGGTGCTCATCTACTG	CCTTCACTCTGATTGCGGGAAAG
Pparg	CAGTGGAGACCGCCCAGGCT	GGATGTCCTCGATGGGCTTCACG
Arg1	CATTGGCTTGCGAGACGTAGAC	GCTGAAGGTCTCTTCCATCACC

Table S3. The raw data for all graphs in the manuscript. An Excel file containing the raw data has been uploaded separately.
